# Regulation of Cell Type-Specific Immunity Networks in Arabidopsis Roots^[CC-BY]^

**DOI:** 10.1105/tpc.20.00154

**Published:** 2020-07-22

**Authors:** Charlotte Rich-Griffin, Ruth Eichmann, Marco U. Reitz, Sophie Hermann, Katherine Woolley-Allen, Paul E. Brown, Kate Wiwatdirekkul, Eddi Esteban, Asher Pasha, Karl-Heinz Kogel, Nicholas J. Provart, Sascha Ott, Patrick Schäfer

**Affiliations:** a School of Life Sciences, University of Warwick, Coventry CV4 7AL, United Kingdom; b Institute of Molecular Botany, Ulm University, 89069 Ulm, Germany; c Institute of Phytopathology, Justus Liebig University, 35392 Giessen, Germany; d Bioinformatics Research Technology Platform, University of Warwick, Coventry CV4 7AL, United Kingdom; e Department of Computer Science, University of Warwick, Coventry CV4 7AL, United Kingdom; f Department of Cell and Systems Biology/Centre for the Analysis of Genome Evolution and Function, University of Toronto, Toronto, Ontario M5S 3B2, Canada; g Warwick Integrative Synthetic Biology Centre, University of Warwick, Coventry CV4 7AL, United Kingdom

## Abstract

While root diseases are among the most devastating stresses in global crop production, our understanding of root immunity is still limited relative to our knowledge of immune responses in leaves. Considering that root performance is based on the concerted functions of its different cell types, we undertook a cell type-specific transcriptome analysis to identify gene networks activated in epidermis, cortex, and pericycle cells of Arabidopsis (*Arabidopsis thaliana*) roots challenged with two immunity elicitors, the bacterial flagellin-derived flg22 and the endogenous Pep1 peptide. Our analyses revealed distinct immunity gene networks in each cell type. To further substantiate our understanding of regulatory patterns underlying these cell type-specific immunity networks, we developed a tool to analyze paired transcription factor binding motifs in the promoters of cell type-specific genes. Our study points toward a connection between cell identity and cell type-specific immunity networks that might guide cell types in launching immune response according to the functional capabilities of each cell type.

## Abstract (precis)

Root cell types possess distinct immunity gene networks that are linked to cell identity networks, as revealed by cell type-specific RNA-seq and a paired motif enrichment tool for promoter analyses.

## INTRODUCTION

Plant roots are essential for plant health and development. In addition to anchoring plants, roots take up nutrients and water and provide protection from soil-based microbes. Conducting these different tasks is especially challenging under changing environments and acute stress conditions. Roots have evolved complex tissues comprising a diversity of cell types with different functions. Organized in concentric layers, Arabidopsis (*Arabidopsis thaliana*) roots consist of an outermost epidermis, followed by cortex, endodermis, pericycle, and root vascular tissue containing xylem and phloem cells (Dolan et al., 1993; Brady et al., 2007). This organization is implemented by the stem cell niche in the very root tip where cell fate is determined, and cell types maintain their given identity throughout their lifetime (van den Berg et al., 1995; Sabatini et al., 2003; Wendrich et al., 2017). Cell types nevertheless possess some plasticity, as exemplified for founder cells that originate from xylem-pole pericycle cell files that initiate lateral root formation (De Smet, 2012; Du and Scheres, 2018). Recent developments in single-cell transcriptomics have helped to further characterize cell types and to define root development but still cannot provide deep transcriptomic profiles (Birnbaum, 2018; Denyer et al., 2019; Jean-Baptiste et al., 2019; Ryu et al., 2019; Zhang et al., 2019; Rich-Griffin et al., 2020). Studies of cell type-specific transcriptomics based on fluorescence-activated cell sorting (FACS), in turn, have significantly advanced our knowledge of the individuality of cell type function in regulating root integrity under changing environments (Birnbaum et al., 2003, 2005; Dinneny et al., 2008; Gifford et al., 2008, 2013; Bargmann et al., 2013; Geng et al., 2013; Walker et al., 2017). While these studies revealed the importance of a coordinated regulation of cell type-specific gene networks to master root development and secure overall root functionality (e.g., growth) under abiotic stress, the function of root cell types in regulating root immunity remains elusive.

**Figure:**
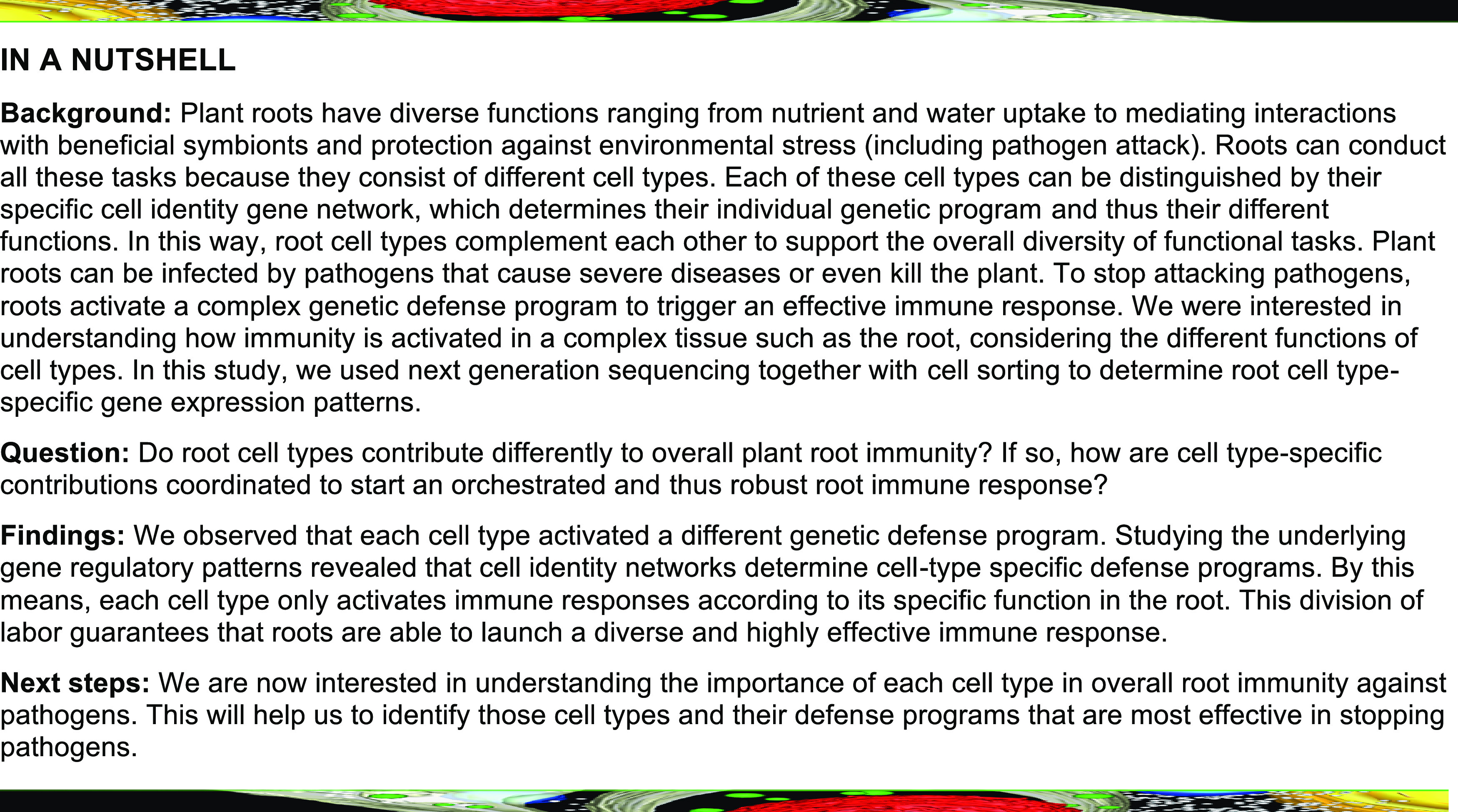


The surface of leaves and roots is the habitat of complex microbiomes consisting of 10^6^ to 10^9^ microbes (per cm^2^ of leaf area or g of soil, respectively); among them are pathogens with lifestyles ranging from biotrophy (life cycle completed on living cells) to necrotrophy (killing cells to complete the life cycle; Berendsen et al., 2012; Bulgarelli et al., 2013; Lareen et al., 2016). Root diseases represent a major threat to crop production, and enhancing root resistance against pathogens by improving processes regulating pattern-triggered immunity (PTI) is an altogether untapped approach to sustain food security (Gewin, 2010; Alexandratos and Bruinsma, 2012; Delgado-Baquerizo et al., 2020; Panth et al., 2020). Plasma membrane-localized pattern recognition receptors (PRRs), to date primarily characterized in leaves, recognize microbe-associated molecular patterns (MAMPs) as nonself molecules from microbes to induce PTI (Jones and Dangl, 2006; Boller and Felix, 2009; Cook et al., 2015). Recognition of the archetypal MAMP flg22 (the active epitope of bacterial flagellin) by the PRR FLAGELLIN-SENSITIVE2 (FLS2) activates PTI responses, including the rapid production of reactive oxygen species (ROS), MITOGEN-ACTIVATED PROTEIN KINASE (MAPK) phosphorylation, and the induction of immunity genes to restrict pathogen infection (Felix et al., 1999; Gómez-Gómez et al., 1999; Asai et al., 2002; Zipfel et al., 2004). Similarly, root cells can recognize flg22 via FLS2 to trigger effective PTI (Millet et al., 2010; Jacobs et al., 2011; Beck et al., 2014; Wyrsch et al., 2015; Poncini et al., 2017; Stringlis et al., 2018). In addition to MAMPs, plants produce damage-associated molecular patterns (DAMPs) in response to pathogens, which are recognized by PRRs as well. Pep1, one of the best studied DAMPs produced in Arabidopsis, is encoded by *PROPEP1* and recognized by the plasma membrane-localized PEP RECEPTOR1 (PEPR1) and PEPR2, triggering similar PTI responses as flg22 (Huffaker et al., 2006; Krol et al., 2010; Yamaguchi et al., 2010; Flury et al., 2013). The PEPR1/2 and FLS2 pathways share common signaling components such as MAPKs (Schulze et al., 2010; Liu et al., 2013; Yamada et al., 2016) but retain certain key differences that might be at least partially explained by additional activities of PEPRs. Qi et al. (2010) identified a unique guanylyl cyclase activity for PEPR1 mediating apoplastic Ca^2+^ influx upon Pep recognition. PEPR1/2-triggered immune signaling was further shown to maintain PTI in plants impaired in MAMP perception and signaling (Tintor et al., 2013; Yamada et al., 2016). Thus, there are clear differences and interdependencies between flg22 and Pep1-induced PTI in Arabidopsis. Nevertheless, the gene networks and underlying regulatory patterns defining DAMP and MAMP-mediated PTI in roots are currently unknown.

Motivated by recent findings suggesting distinct competences of different root cell types in launching PTI (Wyrsch et al., 2015), we wanted to know if flg22 and Pep1 trigger different transcriptional networks in three Arabidopsis root cell types, epidermis, cortex, and pericycle, and if so, whether it would be possible to identify distinct cell type-specific regulatory patterns. Our study demonstrated that very distinct immunity gene networks are activated in the three cell types. Considering that homomeric or heteromeric tandems of transcription factors (TFs) are often sufficient to determine regulatory specificity in eukaryotic cells (Halfon et al., 2000; Vandepoele et al., 2006; Junion et al., 2012; Ezer et al., 2014), we conducted combinatorial TF binding motif analyses to explain the regulatory patterns of cell type-specific gene networks. More specifically, by developing a statistical test for the enrichment of paired TF motifs that accounted for a multiplicity of TF binding sites, we were able to explain cell type-specific differences of Pep1- and flg22-elicited immune networks by specific TF motif combinations. Moreover, our study suggested the importance of cell identity in determining cell type-specific immunity networks. We discuss the significance of such a regulatory connection in specifying cell type functionality and, thus, in securing root integrity under conditions of environmental stress.

## RESULTS

### flg22 and Pep1 Activate Root Immunity through Partially Nonoverlapping Signaling Pathways

Treating Arabidopsis roots with the immunity elicitor flg22 or Pep1 induces PTI responses (e.g., ROS burst, MAPK phosphorylation, induction of PTI marker genes, and eventually inhibited plant growth; Supplemental Figures 1A to 1F). flg22 and Pep1 have been shown to act through overlapping pathways (Krol et al., 2010; Yamaguchi and Huffaker, 2011; Tintor et al., 2013). We previously demonstrated that the beneficial root endophyte *Serendipita indica* (formerly *Piriformospora indica*) suppresses PTI to facilitate root colonization and that flg22 treatment of roots inhibits *S. indica* colonization (Jacobs et al., 2011). In colonized Arabidopsis roots, this fungus inhibits MAPK phosphorylation, PTI marker gene induction, and growth inhibition after flg22 (Supplemental Figures 1B, 1C, and 1E) but not Pep1 treatment (Supplemental Figures 1A to 1C and 1E). Consistent with an effective Pep1-induced immunity, *S. indica* showed improved root colonization of the Pep1 receptor mutant *pepr1 pepr2* (Supplemental Figure 1F). These data suggest that flg22 and Pep1 recruit, at least partially, different signaling pathways to activate PTI in roots.

**Figure 1.**
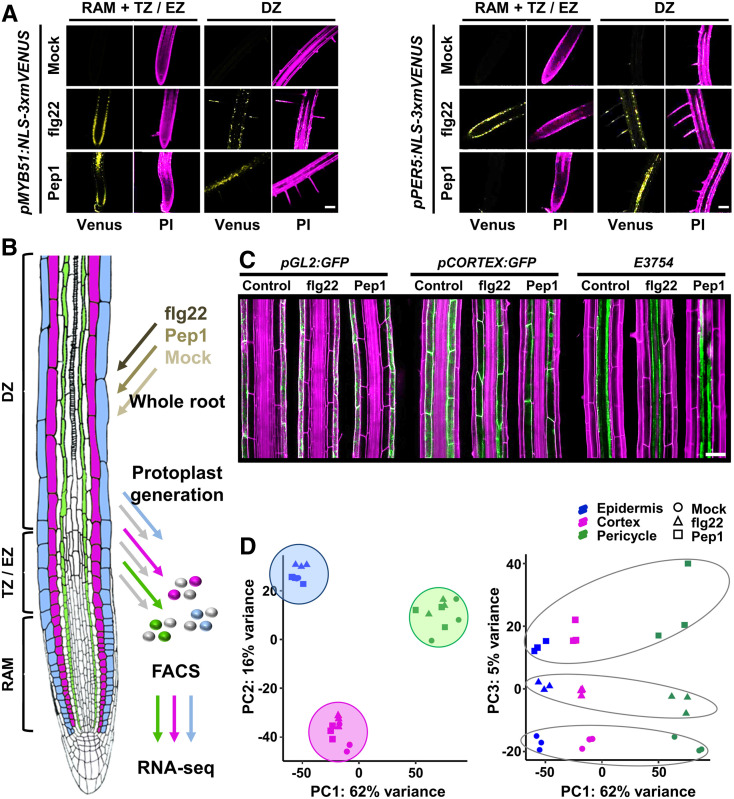
Measurement of Transcriptomic Responses to Immune Elicitors in Three Root Cell Types. **(A)** flg22 and Pep1 activate PTI reporter genes in cells of the RAM with TZ, elongation zone (EZ), and differentiation zone (DZ). PI, propidium iodide. **(B)** Schematic showing the experimental design of this study. For more details, see Supplemental Figure 3. **(C)** Confocal images demonstrating that flg22 and Pep1 do not affect cell type-specific expression of markers *pGL2*:*GFP* (epidermis, atrichoblast), *pCORTEX*:*GFP* (cortex), and *E3754* (xylem-pole pericycle) used for FACS. **(D)** Principal component (PC) analysis of all replicates for each cell type and treatment comparing PC1 and PC2 (left) and PC1 and PC3 (right). All data were based on two **(A)** or three (**[B]** to **[D]**) biological experiments with *n* ≥ 14 (**[A]** and **[C]**) and *n* ≥ 15,000 (**[B]** and **[D]**) plants per experiment and treatment. Bars in **(A)** and **(C)** = 50 µm.

To further explore if PTI can be activated across different root zones, we treated Arabidopsis lines (Poncini et al., 2017) expressing PTI marker gene promoters fused to nucleus-localized mVENUS (*MYB DOMAIN PROTEIN51*, *pMYB51*:*NLS-3xmVENUS*; *PEROXIDASE5*, *pPER5*:*NLS-3xmVENUS*) with flg22 and Pep1. The analyses were conducted to exclude gene induction by other stresses (e.g., wounding). In contrast to recent reports using the same PTI marker lines (Zhou et al., 2020), both elicitors induced all markers in the root apical meristem (RAM), transition zone (TZ), elongation zone, and differentiation zone of roots grown on ATS medium and to a much lesser degree on half-strength Murashige and Skoog (MS) medium (except for *pPER5*:*NLS-3xmVENUS* in RAM/TZ by Pep1; Figure 1A; Supplemental Figures 2A and 2B). Consistently, root growth inhibition was stronger in flg22- or Pep1-treated plants grown on ATS medium (Supplemental Figures 2C and 2D). These findings indicate some extent of PTI suppression, likely because the MES-based buffer system commonly used in MS medium (but not in ATS medium) interfered with the well-known induction of pH changes in response to MAMP perception (Felix et al., 1999). As a result of this MS medium-based PTI quenching effect, all subsequent experiments were done with plants grown on ATS medium.

### Root Cell Types Differ in Their Immunity Gene Networks

Considering the diverse functions of root cell types in root development (Birnbaum et al., 2003; Brady et al., 2007; Gifford et al., 2013) and abiotic stress signaling (Dinneny et al., 2008; Geng et al., 2013), we explored to what extent flg22 and Pep1 affected gene networks in different root cell types. For our studies, we used Arabidopsis lines specifically expressing GFP in epidermis (atrichoblast, *pGL2*:*GFP*), cortex (*pCORTEX*:*GFP*), or pericycle (xylem pole, *E3754*; Masucci et al., 1996; Brady et al., 2007; Gifford et al., 2008; Bargmann et al., 2013; Lin et al., 2015) and treated the roots of ∼15,000 seedlings (per biological repeat) per line with either flg22, Pep1, or mock (Figure 1B; Supplemental Figures 3A and 3B). We selected these cell types due to the importance of the epidermis and cortex (as outer, environment-facing cell layers) in protecting the root against pathogen invasion, while pericycle cells (the outermost cell layer of the inner root tissue and intimately associated with the vasculature) regulate lateral root formation and transport processes and are highly responsive to immune elicitors (Takano et al., 2002; Parizot et al., 2012; Wyrsch et al., 2015; Ross-Elliott et al., 2017). Importantly, previous studies have demonstrated PEPR1/2 and FLS2 function in these cell types and that flg22 and Pep1 reach pericycle cells within minutes after treatment (Wyrsch et al., 2015; Ortiz-Morea et al., 2016). flg22- or Pep1-elicited roots were gently washed to remove flg22 and Pep1 before generating protoplasts from the roots. For each Arabidopsis line and treatment, ∼20,000 GFP-expressing protoplasts were extracted using FACS (Figure 1B; Supplemental Figures 3A and 3B). Importantly, the cell type-specific expression patterns of the marker genes were unchanged in immunity-activated roots (Figure 1C), ensuring uniformity of our isolated cell populations. Similar to cell type transcriptome analyses of abiotic stress networks (Dinneny et al., 2008; Geng et al., 2013), we treated whole roots rather than protoplasts to capture the tissue context of, and intercellular communication between, cell types. In addition, cells were analyzed at 2 h after elicitor treatment to capture early transcriptional changes defining effective PTI and to exclude gene network crosstalk resulting from growth inhibition as a later PTI response (Zipfel et al., 2004).

RNA-seq of FACS-isolated cells (Figure 1B) resulted in ∼315 million reads (∼11.6 million read pairs per library) that were uniquely mapped to gene features in the Arabidopsis genome (Supplemental Table 1; Supplemental Figures 4A to 4F). Principal component (PC) analysis revealed that 82% of the variation was contained within the first three principal components, where PC1 (62% of the variation) separates between cell identity and PC2 (16% of variation) between treatments (Figure 1D; Supplemental Figures 5A and 5B). Cell type marker genes were significantly expressed in the respective cell type populations, indicating that FACS was efficient for cell type isolation (Supplemental Figures 5C to 5E).

**Figure 2.**
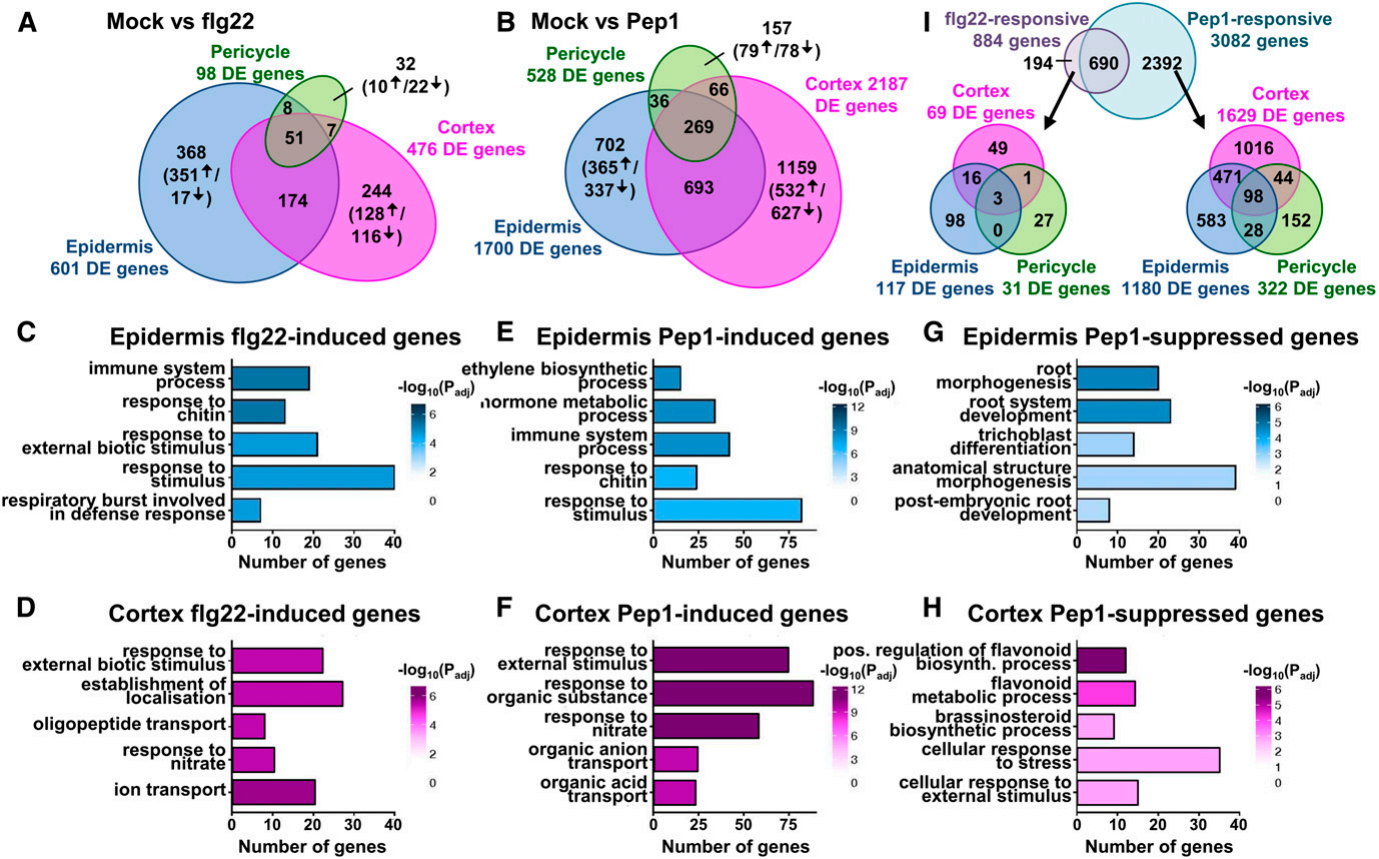
Root Cell Types Differ in Their Immunity Gene Networks. **(A)** and **(B)** Venn diagrams indicating the number of DEGs with cell type-specific expression or expression across cell types upon treatment with flg22 **(A)** or Pep1 **(B)**. Numbers in parentheses indicate upregulated/downregulated DEGs. **(C)** and **(D)** The top 5 nonredundant, most significantly enriched GO terms in genes specifically induced by flg22 in the epidermis **(C)** or cortex **(D)**. In order to make consistent comparisons, the top 128 upregulated genes from each cell type were used to calculate GO enrichment. Tone of color indicates significance degree [log_10_ (P_adj_)]. **(E)** and **(F)** The top 5 nonredundant, most significantly enriched GO terms in genes specifically induced by Pep1 in the epidermis **(E)** or cortex **(F)**. In order to make consistent comparisons, the top 365 upregulated genes from each cell type were used to calculate GO enrichment. Tone of color indicates significance degree [log_10_ (P_adj_)]. **(G)** and **(H)** The top 5 nonredundant, most significantly enriched GO terms in genes specifically suppressed by Pep1 in the epidermis **(G)** or cortex **(H)**. In order to make consistent comparisons, the top 337 downregulated genes from each cell type were used to calculate GO enrichment. Tone of color indicates significance degree [log_10_ (P_adj_)]. **(I)** Venn diagrams show the overlap of flg22- and Pep1-responsive DEG sets aggregated across cell types (top) and the split by cell types for 194 flg22-specific (bottom left) and 2392 Pep1-specific (bottom right) DEGs per cell type.

First, we identified differentially expressed genes (DEGs) in each cell type after flg22 or Pep1 treatment. In total, 3276 unique DEGs responded to one or both elicitors in at least one cell type. Consistent with a recent study (Poncini et al., 2017), Pep1 treatment elicited markedly more DEGs (3082) in roots than flg22 (884). In total, Pep1 resulted in the cell type-specific expression of 702 (epidermis; 365 up/337 down), 1159 (cortex; 532 up/627 down), and 157 (pericycle, 79 up/78 down) DEGs compared with 368 (epidermis; 351 up/17 down), 244 (cortex; 128 up/116 down), and 32 (pericycle; 10 up/22 down) genes with cell type-specific regulation by flg22 (Figures 2A and 2B; Supplemental Table 2; Supplemental Data Sets 1 and 2). Altogether, 65% (2018 genes) of all Pep1-responsive genes and 73% (644 genes) of all flg22-responsive genes (upregulated and downregulated) showed specific expression in only one of the three cell types (Figures 2A and 2B; Supplemental Figure 6; Supplemental Data Set 2). Only 35 genes (e.g., *GLUTATHIONE S-TRANSFERASES* [*GST1/11* and *GSTU12*], *PEROXIDASES* [*PER4/5/61* and *PRX71*], and *INDOLE GLUCOSINOLATE O-METHYLTRANSFERASE*S [*IGMT2/3/4*]) were expressed across all cell types upon flg22 or Pep1 treatment (Supplemental Figure 6; Supplemental Tables 3 and 4). Pericycle replicates were less consistent and noisier, potentially reducing the number of DEGs observed (Supplemental Figure 4; Supplemental Table 2).

To determine if the cell type specificity of flg22- and Pep1-responsive gene networks reflects specific functions, we conducted Gene Ontology (GO) analyses. In the epidermis and cortex, flg22- and Pep1-induced genes were enriched in immunity-associated terms (Figures 2C to 2F). In total, 21% of epidermis-specific (e.g., *NDR1/HIN1-LIKE10* [*NHL10*], *MPK5*, *CHITINASE CLASS IV* [*ATCHITIV*]) and 22.5% of cortex-specific (e.g., *CHITIN ELICITOR RECEPTOR KINASE1* [*CERK1*], *WALL ASSOCIATED KINASE-LIKE2* [*WAKL2*], *WRKY DNA BINDING PROTEIN8* [*WRKY8*]) flg22-responsive genes were associated with immunity terms. Pep1 induced similar proportions of, but distinct, genes associated with immunity: 18% in the epidermis (e.g., *WRKY33*, *FLG22-INDUCED RECEPTOR-LIKE KINASE1* [*FRK1*]) and 19% in the cortex (e.g., *BAK1-INTERACTING RECEPTOR-LIKE KINASE1* [*BIR1*], *WRKY22*, *ARABIDOPSIS NAC DOMAIN CONTAINING PROTEIN19* [*ANAC019*]). The GO term analysis revealed functional specificity; notably, immune and hormone responses were more pronounced in flg22- or Pep1-treated epidermal cells (e.g., immune system process, hormone metabolic process; Figures 2C and 2E). In turn, flg22- and Pep1-induced genes in cortex cells were significantly enriched for terms associated with transport processes (e.g., establishment of localization, oligopeptide transport, organic anion transport; Figures 2D and 2F; Supplemental Data Set 3). Due to the small number of genes (140 genes across all cell types), we were unable to conduct GO analyses for flg22-suppressed genes. However, GO terms associated with Pep1-repressed genes were enriched in terms associated with developmental processes in the epidermis (e.g., root morphogenesis, root system development; Figure 2G) and in flavonoid metabolism and growth hormone synthesis in the cortex (e.g., positive regulation of flavonoid, brassinosteroid biosynthetic process; Figure 2H).

### flg22-Regulated Genes Are Largely Encompassed within a More Diverse Pep1 Gene Network

We next determined the commonalities of flg22 and Pep1 responses between treatments and across cell types (Figure 2I). Aggregating across cell types, 78% (690 of 884 DEGs) of flg22-responsive genes were also regulated by Pep1, whereas 22% (194 of 884 DEGs) were specific to flg22 (Figure 2I). Of the 194 flg22-specific DEGs, 89% (174 DEGs) were only expressed in one cell type (DEGs: 98 in epidermis, 49 in cortex, 27 in pericycle; Figure 2I; Supplemental Data Set 4). The majority of Pep1-responsive genes were specific to Pep1 (2392 of 3082 DEGs; 77%). These Pep1-specific genes were also largely cell type-specific, with 1016 DEGs expressed in the cortex, 583 in epidermal cells, and 152 in pericycle cells (Figure 2I; Supplemental Data Set 4). GO analyses of these elicitor-specific DEGs revealed that flg22-responsive genes in epidermal and cortical cells were enriched in GO terms defining transport processes (e.g., organic acid transport, amino acid transport, nitrate transport), whereas Pep1-enriched terms were associated with hormone metabolism and signaling (e.g., hormone metabolic process, salicylic acid biosynthetic process) in these two cell types (Supplemental Data Set 5). Overall, our results show that both flg22 and Pep1 activate distinct gene networks in each cell type and that the flg22 response is largely encompassed within a much more diverse Pep1 response.

### Immunity Networks Only Partially Overlap with Cell Identity Gene Networks

**Figure 3.**
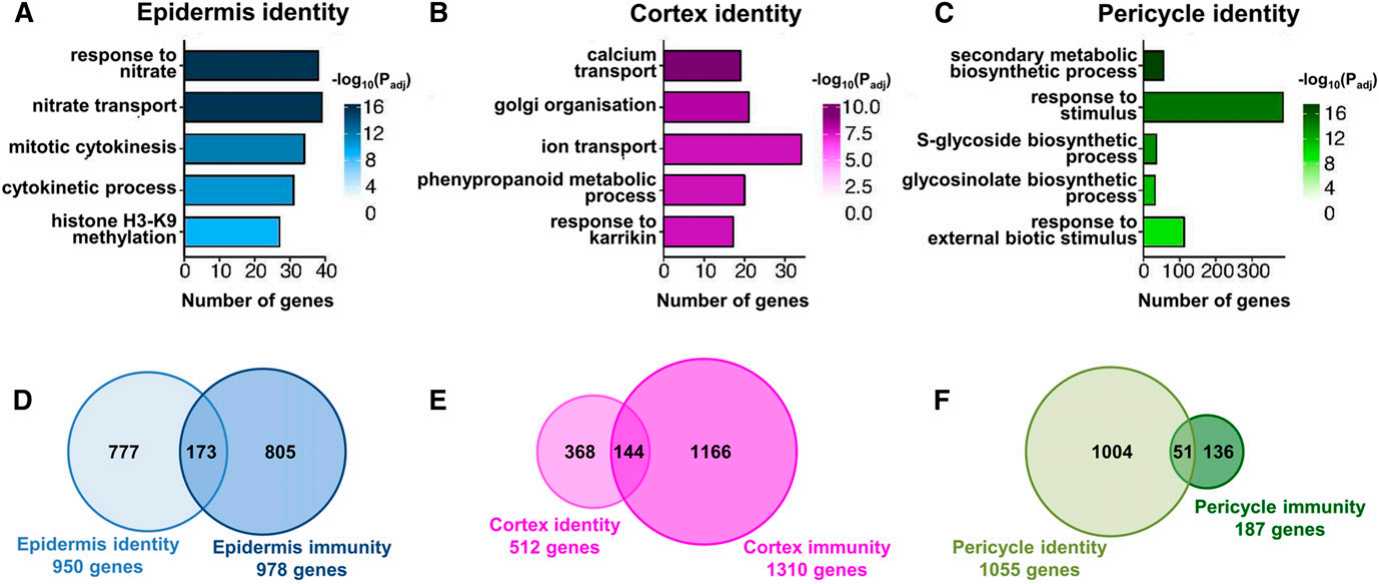
Overlap of Cell Type-Specific Immunity and Cell Identity Gene Networks. **(A)** to **(C)** The top 5 nonredundant, most significantly enriched GO terms in cell type-specific identity genes. The 512 most significant upregulated genes from each cell type were used for consistent comparisons across cell types. Tone of color indicates significance degree [log_10_ (P_adj_)]. **(D)** to **(F)** Venn diagrams showing the overlap between cell type-specific identity genes and cell type-specific, flg22- or Pep1-reponsive immunity genes.

Our observation that Pep1-repressed genes function in root growth and development (Figure 2G) is in agreement with the reported root growth-inhibiting effect of PTI (Gómez-Gómez et al., 1999; Jacobs et al., 2011). It is known that the maintenance of the identity of each cell type, which is defined by cell type-specific functions, is essential for overall root integrity and root growth, especially under stress (Iyer-Pascuzzi et al., 2011; Geng et al., 2013). To determine if PTI affects cell (type) identity networks, we first defined the cell identity-specific transcriptomes (using our RNA-seq data from mock-treated samples) and identified 950 genes as specifically enriched in epidermis, 512 in cortex, and 1055 in pericycle (Supplemental Data Set 6). These enriched data sets were confirmed to strongly overlap (P < 10^−6^, Fisher’s exact test) with published cell identity gene sets (Bargmann et al., 2013), and distinct GO terms were associated with each set of identity genes specifying the different functions of each cell type (Figures 3A to 3C; Supplemental Data Set 6). By comparing cell identity with cell type-specific PTI gene sets (by combining flg22 and Pep1 DEGs per cell type), we found that PTI affected 18% (epidermis), 28% (cortex), and 5% (pericycle) of respective cell identity genes (Figures 3D to 3F; Supplemental Data Set 7). Similarly, salt stress or iron deprivation-regulated networks overlapped with cell identity networks, which was found to support cell type-specific responses to abiotic stresses (Dinneny et al., 2008; Iyer-Pascuzzi et al., 2011). We therefore wanted to understand how cell identity and immunity networks are linked.

### Specific TF Pairing Links Cell Identity with Cell Type-Specific Immunity Networks

**Figure 4.**
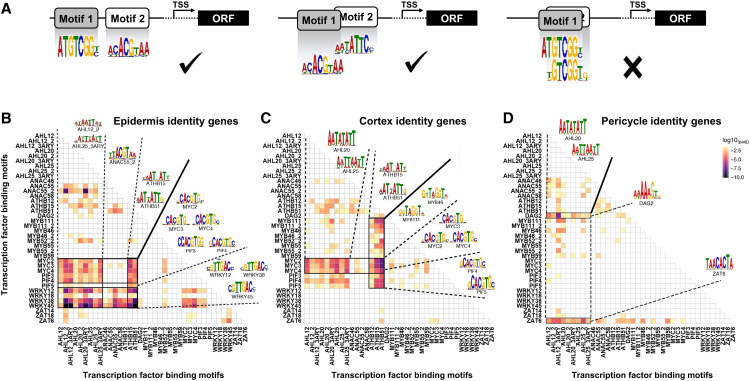
Different Cell Types Show Distinct Patterns of Paired-Motif Enrichment in Cell Type-Specific Genes. **(A)** Diagram illustrating the acceptance and rejection of overlaps of TF binding motifs in the paired-motif enrichment analysis. ORF, open reading frame; TSS, transcriptional start site. **(B)** to **(D)** Heatmaps representing P values (log10_Padj_) for enrichment of motif pairs in epidermis **(B)**, cortex **(C)**, and pericycle **(D)** identity genes. *x* and *y* axes of all heatmaps show the same order of TF binding motifs. Pixel colors indicate significance values (log10_Padj_) for the abundance of given motif pairs in the promoters of identity genes per cell type.

To regulate gene networks, TFs exert their activity at their site (cell type) of synthesis but can also move across cell boundaries. For instance, fundamental root developmental processes such as root patterning, cell fate decision, and root growth depend on the mobility of TFs such as SHORTROOT, KNOTTED1, and PHLOEM EARLY DOF (Nakajima et al., 2001; Xu et al., 2011; Clark et al., 2016; Miyashima et al., 2019). We therefore analyzed the presence and abundance of TF binding motifs in the promoters of DEGs to identify cell type-specific gene regulatory patterns and reveal any interdependencies between cell identity and cell type-specific immunity. Based on the clear evidence that combinatorial TF pairing is most crucial in controlling gene expression (Achard et al., 2009; Van de Velde et al., 2014; Lewis et al., 2015), we developed the Paired Motif Enrichment Tool (PMET) method to identify pairs of TF binding motifs within the promoters of our cell identity and cell type-specific DEG sets based on the following criteria, as depicted in Figure 4A: (1) a multiplicity of TF binding motifs in each promoter, and (2) acceptance of limited but (3) rejection of major overlapping of binding sites with (4) high motif specificities (Figure 4A; see Methods). By tolerating limited motif overlap, our analysis allowed us to consider TF complexes associated with compound binding sites (Rodríguez-Martínez et al., 2017).

In the first step, we identified enriched motif pairs in our cell identity gene networks using a TF binding motif database derived from the work of Franco-Zorrilla et al. (2014). To enable direct comparisons with equal statistical power, we balanced the data sets in that we compared the promoters of all 512 (368 + 144 genes; Figure 3E) cortex identity genes with the promoters of the 512 most significantly upregulated epidermis and pericycle identity genes (Figures 3D to 3F). We found highly significant enrichment of a number of motif pairs within the promoters of 472 epidermis (92%), 442 cortex (86%), and 461 pericycle (90%) cell identity genes (Figures 4B to 4D; Supplemental Data Set 8). Comparing epidermis, cortex, and pericycle, the pattern of enriched motif combinations was distinctive between all cell types. For instance, within the promoters of epidermis identity genes, motifs for four different WRKY TFs (WRKY12/38/45 and to a lesser extent WRKY18) were found to pair uniquely with a wide range of motifs (Figure 4B). In particular, WRKYs were enriched with binding motifs for AT-HOOK MOTIF CONTAINING NUCLEAR LOCALIZED (AHL), *ARABIDOPSIS THALIANA* HOMEOBOX (ATHB), and ARABIDOPSIS NAC (ANAC) TFs within the promoters of 40% of all epidermis identity genes (203 of 512 genes). In the cortex gene promoters, we observed specific pairing of ATHB with MYB TF binding motifs, whereas pairing between AHLs and DOF AFFECTING GERMINATION (DAG2) or ZINC FINGER OF ARABIDOPSIS6 (ZAT6) was pericycle-specific (Figures 4C and 4D). We also noted some overlap in enriched motif pairs between epidermis and cortex identity networks, with MYCs and PHYTOCHROME-INTERACTING FACTORs (PIFs) showing pairing with AHL and ATHB motifs for both cell types (Figures 4B and 4C).

Interestingly, we observed the distinct pairing of stress- and development-associated TFs in all cell types. In the epidermis, for instance, AHL and ATHB TFs, which function in growth and development (Matsushita et al., 2007; Hur et al., 2015; Miao et al., 2018), paired with WRKYs, a large family of Arabidopsis TFs (>70 members) with regulatory functions in plant innate immunity and abiotic stress responses (Pandey and Somssich, 2009; Rushton et al., 2010), whereas in the cortex, AHLs and ATHBs paired with plant immune/jasmonate-responsive MYC2/3/4 TFs (Fernández-Calvo et al., 2011; Schweizer et al., 2013). Based on this apparent connection between developmental and stress networks within identity genes, we analyzed cell type-specific flg22- and Pep1-responsive promoter sets to test whether this connection was also present following immune activation. We had to exclude pericycle data (Figures 2A and 2B) from these analyses due to the low number of flg22- or Pep1-responsive genes. To make the enrichment scores across treated cell types comparable, we again equalized gene set sizes (see Methods).

**Figure 5.**
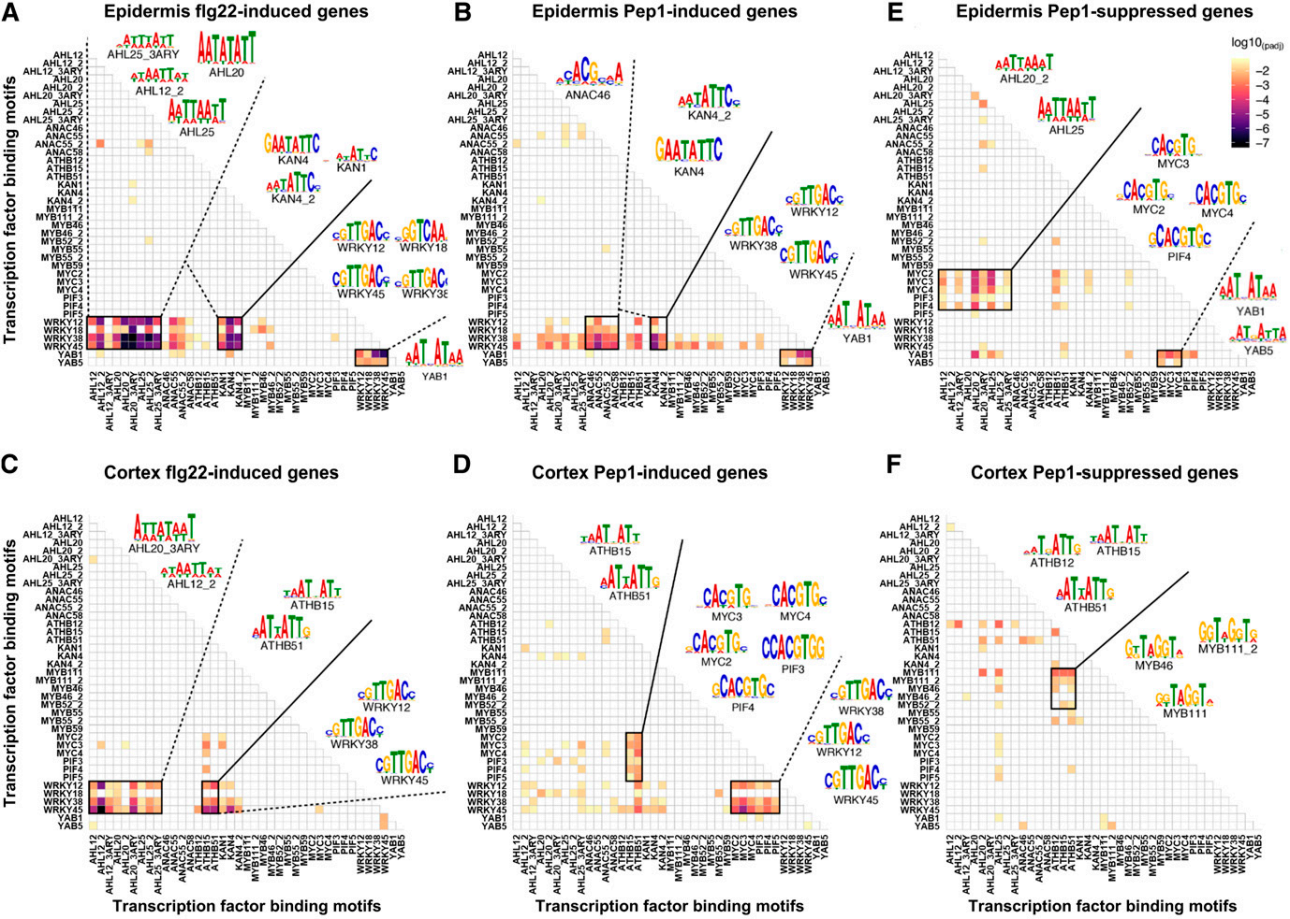
Paired-Motif Enrichment of Cell Type-Specific flg22- and Pep1-Responsive Genes. Heatmaps represent P values (log10_Padj_) for enrichment of motif pairs in the promoters of flg22-induced epidermis **(A)** and cortex genes **(B)**, promoters of Pep1-induced epidermis **(C)** and cortex genes **(D)**, and promoters of Pep1-suppressed epidermis **(E)** and cortex genes **(F)**. *x* and *y* axes of all heatmaps show the same order of TF binding motifs. Pixel colors indicate significance values (log10_Padj_) for the abundance of given motif pairs in the promoters of identity genes per cell type.

The sensitivity of the promoter analysis tool allowed us to detect elicitor-specific changes in motif pairing in each cell type. For the epidermis, a highly significant pairing of WRKY12/18/36/45 and AHL12/20/25 motifs was identified as specific for flg22-induced genes (Figure 5A), in contrast to the enriched pairing of WRKY12/18/36/45 and ANAC (ANAC46/55/55_2/58) motifs in the DEG promoters of Pep1-treated epidermal cells (Figure 5B). In the epidermis, there was also a common enriched pairing of WRKY12/18/38/45 with YABBY1 (YAB1) and YAB5 as well as with KANADI 1 (KAN1) and KAN4 motifs between flg22- and Pep1-induced genes (Figures 5A and 5B; Supplemental Data Sets 9 and 10). YABBYs participate in lateral organ and meristem development (Sarojam et al., 2010), while KANADI family member acts as negative regulators of embryo development (McAbee et al., 2006), root development (Hawker and Bowman, 2004), and vascular tissue formation (Ilegems et al., 2010). For the cortex, we detected enriched pairing of WRKY12/38/45 with ATHB15/51 and AHL12/20 motifs in the promoters of flg22-induced genes (Figure 5C). The promoters of Pep1-induced cortical genes, in turn, were dominated by MYC2/3/4 and PIF3/4/5 motif pairing with WRKY12/38/45 and ATHB15/51 (Figure 5D).

In summary, the analyses again revealed the interaction of stress- and development-associated TFs as a consistent theme, irrespective of flg22, Pep1, or cell type. Interestingly, in the promoters of Pep1-repressed genes, which were enriched in developmental GO terms (Figures 2G and 2H), we detected a higher abundance of developmental TF motif pairing (with a complete absence of WRKY motifs). While Pep1-suppressed genes in the epidermis showed an enriched pairing of MYC2/3/4 and PIF3/4 with AHL12/20/25 and YAB1 motifs, the promoters of Pep1-suppressed cortical genes revealed ATHB12/15/51 motifs paired with MYB46/52/111. Our paired-motif enrichment analyses thus revealed a pairing of stress- and development-associated TFs in regulating immunity networks in a cell type-specific manner. Overall, for flg22, our promoter analyses uncovered an enrichment of highly specific motif pairs in the promoters of 90% of flg22-induced epidermis genes and 86% of cortex genes (Figures 5A and 5C). For Pep1, we identified paired-motif enrichment in the promoters of 58 and 60% of epidermis- or cortex-induced genes, respectively (Figures 5B and 5D) as well as 57 and 50% of epidermis- or cortex-suppressed genes, respectively (Figures 5E and 5F; Supplemental Data Set 10). Despite some overlap, the promoters of DEGs specifically regulated by Pep1 and flg22 generally showed clear differences in motif enrichment within and across cell types.

### TF Binding Motif Pairs Determine Cell Type-Specific and Elicitor-Responsive Gene Regulation

**Figure 6.**
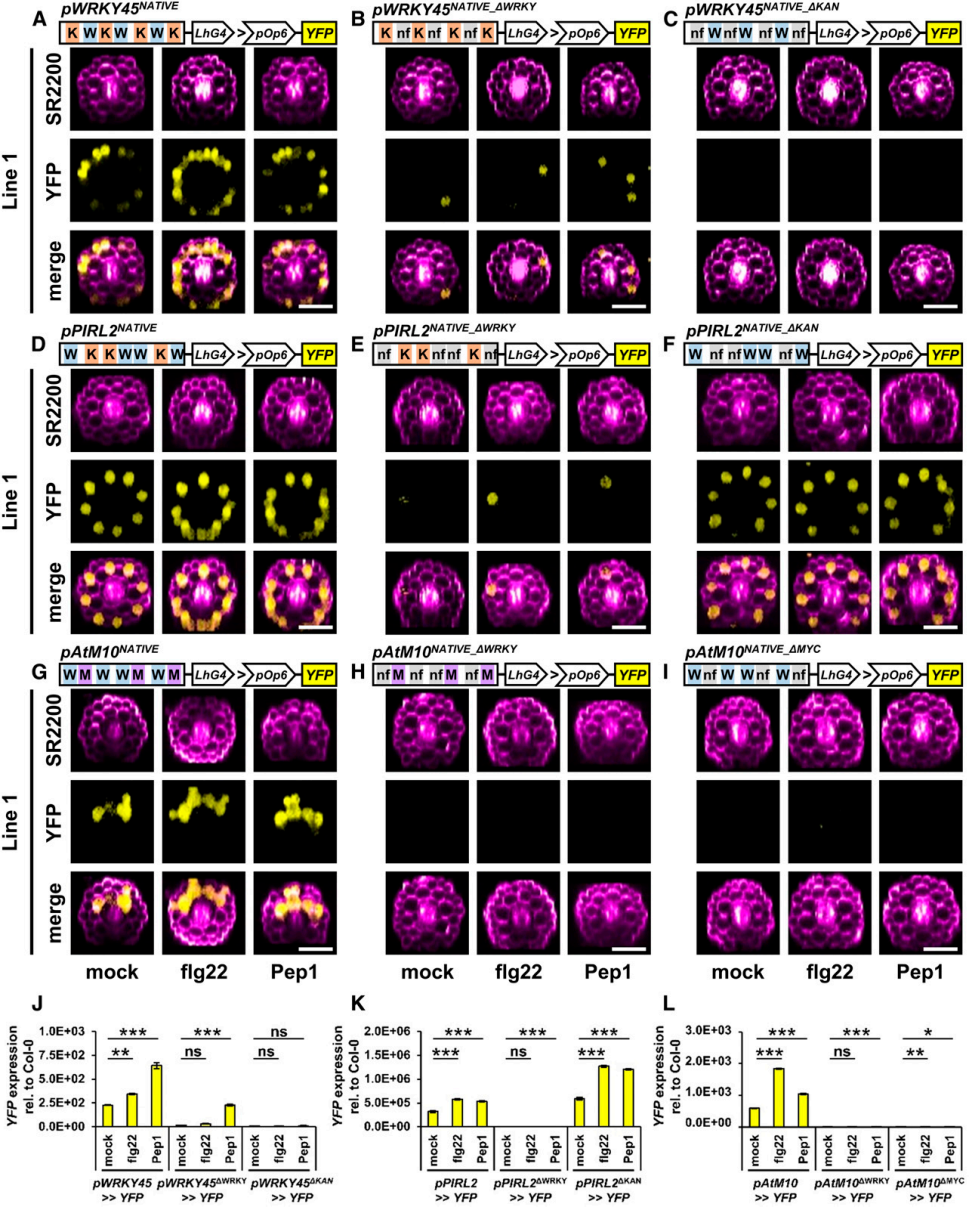
Predicted Promoter Motif Pairs in Native Promoters Are Essential for Cell Type-Specific Gene Regulation in Planta. In **(A)** to **(I)**, native promoters with or without deletions in predicted TF binding motifs were analyzed in two independent plant lines stably expressing the *Promoter*:*LhG4* > *pOp6*:*YFP* transactivation system. Please see the results for both lines per construct in Supplemental Figure 8. SCRI Renaissance 2200 (SR2200) was used to stain root cells. **(A)** to **(C)** and **(J)** WRKY (W) and KANADI (K) motifs in the native promoter of *WRKY45* specify epidermis-specific *YFP* expression, as indicated by confocal microscopy of axial root sections of transformed Arabidopsis lines (**[A]** to **[C]**). qRT-PCR-based quantification of flg22/Pep1 responsiveness in the native promoter lines shown in **(A)** to **(C)** is shown in **(J)**. nf, nonfunctional sequence. **(D)** to **(F)** and **(K)** WRKY and KANADI motifs in the native promoter of *PIRL2* specify epidermis-specific *YFP* expression, as indicated by confocal microscopy of axial root sections of transformed Arabidopsis lines (**[D]** to **[F]**). qRT-PCR-based quantification of flg22/Pep1 responsiveness in the native promoter lines shown in **(D)** to **(F)** is shown in **(K)**. In the case of the *PIRL2* promoter, the WRKY motifs were sufficient for and enhanced cell type-specific and elicitor-induced *YFP* expression, as shown in **(H)**. **(G)** to **(I)** and **(L)** WRKY and MYC (M) motifs in the native promoter of *AtM10* specify cortex-enriched *YFP* expression, as indicated by confocal microscopy of axial root sections of transformed Arabidopsis lines (**[G]** to **[I]**). qRT-PCR-based quantification of flg22/Pep1 responsiveness in the native promoter lines shown in **(G)** to **(I)** is shown in **(L)**. The qRT-PCR data in **(J)** to **(L)** are based on one representative biological experiment for one line. Asterisks indicate significant differences at P < 0.05 (*), P < 0.01 (**), and P < 0.001 (***) according to one-way ANOVA and Bonferroni posthoc test. ns, not significant. All other data are based on three biological experiments with *n* ≥ 14 plants per experiment and treatment. Bars = 50 µm.

As a final step, we wanted to confirm the accuracy of the predictions from our promoter motif enrichment tool in interpreting cell type-specific expression patterns. We first identified a set of native promoters from genes that showed cell type-specific expression and enrichment for a motif pair. For the epidermis, *WRKY45* and *PLANT INTRACELLULAR RAS GROUP-RELATED LRR2* (*PIRL2*) showed specific expression and predicted KAN-WRKY pairs. *AtM10* and *BASIC HELIX-LOOP-HELIX92* (*bHLH92*) were chosen as cortex-specific genes enriched in MYC-WRKY pairs in their promoters. For our analyses we used the *pPROMOTER*:*LhG4* > *pOp6*:*YFP* transactivation system (Moore et al., 1998; Craft et al., 2005; Costa et al., 2014), where our native promoters were fused to *LhG4* (*pPROMOTER*^*NATIVE*^:*LhG4*), which binds to the *Op6* promoter (*pOP6*) to transactivate *yellow fluorescent protein *(*YFP*) expression. For each promoter variant, we identified two independent Arabidopsis lines for further analyses. Excitingly, except for *bHLH92* constructs, where we could not identify any lines, we confirmed the predicted cell type-specific expression patterns in planta: *pOp6*:*YFP* lines expressing *pWRKY45*^*NATIVE*^:*LhG4* or *pPIRL2*^*NATIVE*^:*LhG4* showed epidermis-specific *YFP* expression, whereas *pAtM10*^*NATIVE*^:*LhG4* mediated cortex-enriched *YFP* expression (Figures 6A, 6D, and 6G; Supplemental Figures 7A to 7F and 8A, 8D, and 8G). Moreover, replacing one type of the predicted motifs in the otherwise unaltered native promoters by a nonfunctional sequence (see Methods for design) was sufficient to abolish *YFP* expression (Figures 6B, 6C, 6E, 6F, 6H, and 6I; Supplemental Figures 8B, 8C, 8E, 8F, 8H, and 8I), indicating the accuracy of our prediction tool in identifying motif pairs that determine cell type-specific expression. We further confirmed the predicted function of these motif pairs in cell type-specific regulation by flg22 or Pep1. Again, eliminating one type of motif was sufficient to abolish (or significantly reduce Pep1-induced *YFP* transactivation in plants expressing *pWRKY45*^*NATIVE_ΔWRKY*^) cell type-specific elicitor inducibility (Figures 6J to 6L).

**Figure 7.**
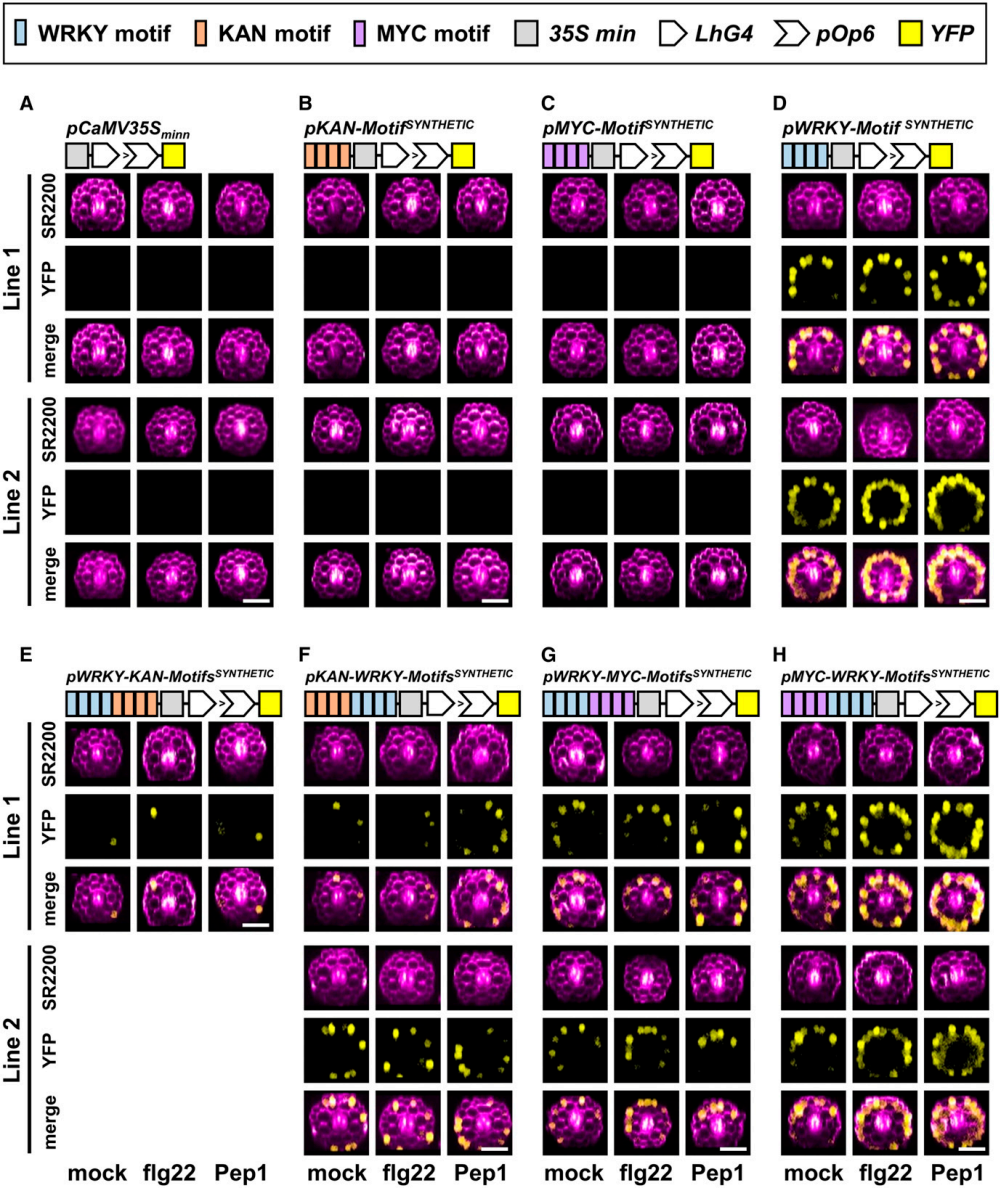
“Motif-Only” Synthetic Promoters Reveal Regulatory Functions of Promoter Motifs in Gene Expression in Planta. Synthetic promoters only consisting of a set of four motifs or motif combinations and a *CaMV35S* minimal promoter (but lacking any native promoter backbone sequence) were analyzed in plant lines stably expressing the *Promoter*:*LhG4* > *pOp6*:*YFP* transactivation system. Except for WRKY-KANADI motif combinations, two independent plant lines were analyzed per promoter construct by confocal microscopy of axial root sections. **(A)** to **(D)** Plant lines expressing the *CaMV35S* minimal promoter **(A)**, 4× KANADI binding motifs **(B)**, or 4× MYC binding motifs **(C)** did not activate *YFP* expression, whereas plant lines expressing 4× WRKY resulted in epidermis-specific *YFP* expression **(D)**. **(E)** and **(F)** Proximal **(E)** and distal **(F)** locations of 4× KANADI binding motifs inhibited 4× WRKY-mediated *YFP* expression. **(G)** and **(H)** Proximal **(G)** but not distal **(H)** locations of 4× MYC binding motifs inhibited 4× WRKY-mediated *YFP* expression. Root cells were stained with SCRI Renaissance 2200 (SR2200). All data were based on two biological experiments with *n* ≥ 12 plants per experiment and treatment. Bars = 50 µm.

In the case of plants expressing *pPIRL2*^*NATIVE_ΔKAN*^ (lacking KAN motifs in the native *PIRL2* promoter), we detected enhanced basal and elicitor-induced, cell type-specific expression of *YFP* (under mock, flg22, or Pep1; Figures 6D to 6F; Supplemental Figures 8D to 8F). This led us to investigate the roles of the identified motifs as activators or repressors of gene transcription. We therefore generated synthetic promoters consisting of motif sequences linked with short nonfunctional linker sequences (see Methods for design) and, to exclude any other regulatory motifs, lacking any promoter backbone sequence. These synthetic promoters were run by a proximally placed minimal *CaMV35S* promoter (*CaMV35S*_*min*_), and plants expressing this minimal *CaMV35S* promoter alone (*pCaMV35S*_*min*_:*LhG4*) did not induce *YFP* expression. As expected, when combining *CaMV35S*_*min*_ with a set of 4× KAN motifs (*pKANADI-Motif*^*SYNTHETIC*^:*LhG4*) or 4× MYC motifs (*pMYC-Motif*^*SYNTHETIC*^:*LhG4*), we did not induce *YFP* expression, whereas the combination with 4× WRKY motifs (*pWRKY-Motif*^*SYNTHETIC*^:*LhG4*) showed epidermis-specific *YFP* expression (Figures 7A to 7D; Supplemental Figures 9A and 9B). This epidermis-specific expression was almost abolished when 4× KAN motifs were placed proximal to 4× WRKY motifs (*pWRKY-KANADI-Motifs*^*SYNTHETIC*^:*LhG4*) and was strongly reduced when arranged distally to 4× WRKY motifs (*pKANADI-WRKY-Motifs*^*SYNTHETIC*^:*LhG4*) under mock, flg22, and Pep1 treatment (Figures 7E and 7F; note that deep screening only revealed one transformed line for *pWRKY-KANADI-Motifs*^*SYNTHETIC*^:*LhG4*). These findings suggest that KAN motifs inhibit 4× WRKY-mediated *YFP* expression and that KAN motifs could be considered to be suppressor elements. However, the suppressor activity was stronger when 4× KAN motifs were placed proximal to WRKY motifs (Figures 7E and 7F; Supplemental Figures 9A and 9B). To validate this positional effect further, we generated a second set of synthetic promoters with 4× WRKY and 4× MYC motifs. When 4× MYC motifs were placed proximally to 4× WRKY motifs (*pWRKY-MYC-Motifs*^*SYNTHETIC*^:*LhG4*), the plants showed reduced *YFP* expression (Figure 7G), whereas full epidermis-specific *YFP* expression levels were detected when 4× MYC motifs had a distal position to 4× WRKY motifs (*pMYC-WRKY-Motifs*^*SYNTHETIC*^:*LhG4*; Figure 7H; Supplemental Figures 9A and 9B). This indicates the importance of motif positions in balancing gene expression and that KAN motifs apparently functioned as repressor elements in our setup.

Taken together, our promoter motif prediction tool identified paired TF binding sites, and we confirmed the importance of the predicted pairs of TF binding motifs in regulating cell type-specific gene expression. This tool is also suitable for identifying relevant promoter motifs whose function can be studied in synthetic promoters as a helpful step in generating customized or minimal synthetic promoters for the targeted analysis of gene network regulation in plants.

## DISCUSSION

The physical inaccessibility of roots impedes the detection and control of soil-borne pathogens and explains the high relevance of root diseases for staple crop production (Gewin, 2010; Alexandratos and Bruinsma, 2012; Panth et al., 2020). Under the projected future global warming, the frequency of root diseases is expected to rise at a global scale (Delgado-Baquerizo et al., 2020). Therefore, new control strategies are needed, which requires a better understanding of the genetic disease resistance potentials and regulatory mechanisms of underlying immune responses in roots. In this study, we analyzed the immunity gene networks of epidermis and cortex cells, which build the outer frontier to the rhizosphere, as well as the pericycle, the “outer frontier” of the inner root vasculature. To obtain a comprehensive picture of root immunity, we analyzed cell type-specific PTI in response to the MAMP flg22, which activates PTI against bacteria, and the DAMP Pep1, a plant-derived PTI elicitor that is activated upon the perception of different microbes and defense hormones and, hence, might trigger the full array of all PTI responses against a larger variety of pathogens (Ryan et al., 2007; Lori et al., 2015). By combining RNA-seq data with PMET, a promoter analysis approach, we were able to identify distinct regulatory patterns within immunity networks activated in a cell type-specific manner.

The analytic design of PMET considers the pairing of TF binding sites on promoters, which proved to be sufficient to explain the regulatory patterns of 50 to 90% of DEGs per cell type and treatment. The application of PMET in the analysis of complex transcriptomes is not restricted to plants but works for eukaryotes in general. We found it equally efficient for elucidating network regulation in neuroinflammatory disorders in mouse (Schang et al., 2018). In this study, PMET revealed the close interplay of cell type-specific immunity with identity networks that are essential for tissue organization and development. Consistent with this finding, cell type-specific transcriptomics revealed a close interaction of abiotic stress with cell identity networks. The adaptation to different abiotic stresses includes a highly coordinated and cell type-specific redirection of gene networks to maintain root function (e.g., growth; Dinneny et al., 2008; Gifford et al., 2008; Iyer-Pascuzzi et al., 2011). In addition, the underlying rebooting of cell type-specific signaling under salt stress involved hormones, such as abscisic acid, to readjust root growth and adapt root system architecture (Geng et al., 2013). Besides hormones, RAPID ALKALINIZATION FACTOR (RALF) peptides in interaction with their receptors THESEUS1 and FERONIA regulate lateral root formation (Haruta et al., 2014; Murphy et al., 2016; Gonneau et al., 2018; Jourquin et al., 2020). Interestingly, RALF-FERONIA complexes also control PTI (Stegmann et al., 2017). These studies suggest a close interdependency of developmental and stress networks at the perception (receptor) to signal transduction level (e.g., hormones) in order to adjust overall root development to changing environments. The observed interplay of cell identity and cell type-specific immune gene networks in our study adds regulatory mechanisms at the gene level to support this crosstalk at the plant development-environment interface. Altogether, these findings demonstrate a remarkable complexity of immunity signaling in roots.

**Figure 8.**
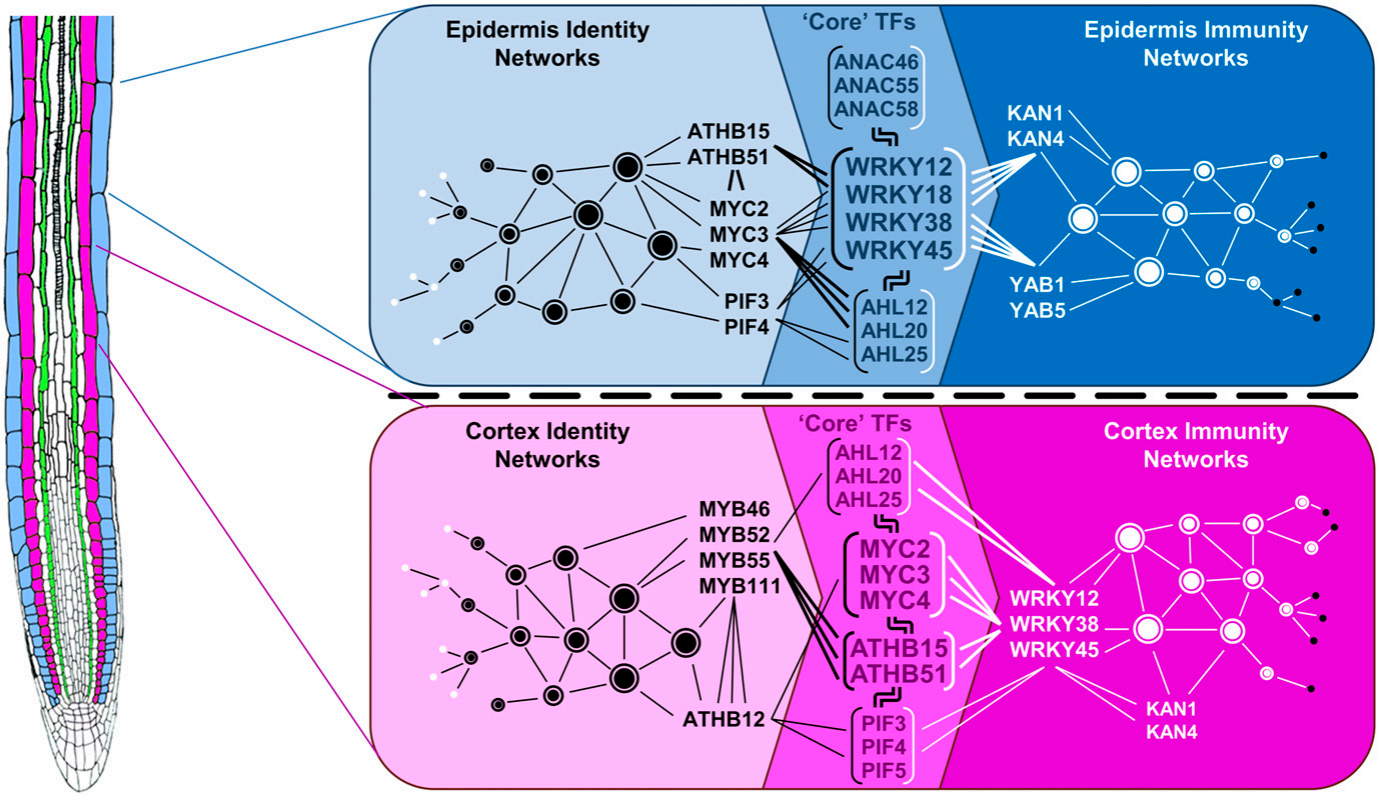
Model Illustrating TF-Mediated Linkage of Cell Identity and Cell Type-Specific Immunity Networks. Based on our paired motif enrichment analyses, TFs can be assigned to cell identity or cell type-specific immunity networks, and immunity networks differ between epidermis and cortex cells. Those TFs that can be found in both (identity and immunity) networks might act as core TFs (bracketed) to connect epidermis identity with cell type-specific immunity networks. Among all core TFs, WRKY12/18/38/45 and MYC2/3/4 together with ATHB15/51 showed the strongest links in the epidermis and cortex, respectively.

Although cell type specificity in immunity gene regulation may require a higher degree of coordination (e.g., numerous regulatory and signaling proteins), it apparently adds to the robustness and flexibility required for a root system to adapt to changing environments. Accordingly, recent studies observed flg22 responsiveness in cell types of different developmental ages, although qualitative differences in the immune competences of root cell types appeared to occur (Beck et al., 2014; Wyrsch et al., 2015; Poncini et al., 2017). Supported by PMET-base analyses, we obtained insights into how this tight coordination of cell type-specific immune responses may be achieved. Our observation that root cell types keep their identity under biotic stress, as indicated by cell type marker expression, principal component analyses, and studying the regulation of cell identity genes under immunity (Figures 1C, 1D, and D to F3D to 3F), might be most critical in this respect. We found that the pairing of TF motifs for DEGs differed depending on the cell type, and we confirmed the significance of TF pairs for cell type-specific expression of immunity genes in our functional promoter analyses (Figure 6; Supplemental Figures 7 and 8). Moreover, our data suggest a model where certain TF combinations prevailed in specific cell types in a treatment-dependent manner and revealed “core” TFs that link cell identity with cell type immunity networks (Figure 8). For the epidermis-specific networks, WRKY12/18/38/45 appear to act as core TFs (together with ANAC46/55/58 and AHL12/20/25) to connect identity and immunity networks by pairing with ATHB, MYC, and PIF TFs of the epidermis identity network and with KAN and YAB TFs of the epidermis immunity network. In the cortex, in turn, MYC2/3/4 and ATHB15/51 (together with PIF3/4/5 and AHL12/20/25) might serve as core TFs that pair with WRKYs to direct cortex-specific immunity as well as with ATHB12 and MYBs to corroborate cortex identity networks. Thus, the cooperation of different TF families in specific combinations underpins the highly cell type-specific immunity networks that we observed.

In addition to defining links between cell identity and immunity networks, our promoter motif prediction tool can disentangle apparently contradictory functions of TFs. We observed a striking difference in the context-dependent association of MYC motifs in Pep1 responses. MYC TFs have been implicated in Pep1-mediated signaling. Pep peptides specifically activate the MYC2-dependent branch of jasmonic acid signaling (Bartels and Boller, 2015), and MYC2 can act as both an activator and a repressor of jasmonic acid-mediated gene expression (Dombrecht et al., 2007). Consistent with this observation, MYC2/3/4 binding motifs were found to pair with WRKY and ATHB motifs in the promoters of Pep1 upregulated cortex genes (Figure 5D). By contrast, MYC2/3/4 motifs paired with AHL12/20/25 motifs in the promoters of downregulated genes in the epidermis (Figure 5E). The analyses thus suggest a potential multifunctionality of specific TF family member combinations, whereby they can act with heterogeneous partners in different cell types to control contrasting transcriptional outputs. In addition, the observed context dependency of regulatory functions may reduce the perceived redundancy among TFs that recognize highly similar sites if investigated in isolation.

By computing the presence of TF binding motif combinations for DEGs in our RNA-seq data, we observed that cell identity and stress-responsive gene networks coexist in each root cell type. As reported for abiotic stress integration (Dinneny et al., 2008; Iyer-Pascuzzi et al., 2011; Geng et al., 2013), our data support the concept that cell identity underpins transcriptional reprogramming leading to cell specificity in response to signal perception, even modulating outputs from strong elicitors such as MAMPs. Linking immunity to cell identity networks would guarantee cell type-specific coordination of immune responses with individual and measured contributions from each cell type. Such a coregulatory model would likely be applicable to root responses to all environmental stresses and add the necessary flexibility to gene regulation (Dinneny et al., 2008; Van de Velde et al., 2014). In the future, it will be interesting to explore to what extent cell identity and core TFs contribute to cell type specificity of immune responses and resistance against pathogens as well as root growth upon the activation of immunity. Our analyses using promoter lines (Figure 6; Supplemental Figures 7 and 8) suggest the feasibility of such functional dissection studies.

## METHODS

### Plant Materials, Growth Conditions, and Treatments

Seeds of Arabidopsis (*Arabidopsis thaliana*) ecotype Col-0 were obtained from the Nottingham Arabidopsis Stock Center. Seeds of the *pepr1-2 pepr2-2* mutant were kindly provided by Y. Yamaguchi (Osaka Prefecture University). Marker lines for the three cell types, *pGL2*:*GFP* (epidermis atrichoblast; Lin et al. 2015), *pCORTEX*:*GFP* (cortex), and *E3754* (xylem-pole pericycle; Bargmann et al. 2013), were obtained from Miriam Gifford (University of Warwick). PTI marker lines *pMYB51*:*NLS-3xmVENUS* and *pPER5*:*NLS-3xmVENUS* were provided by Silke Lehmann (University of Warwick; Poncini et al., 2017). Plants were grown on vertical square Petri dishes on ATS medium (Lincoln et al., 1990) without sucrose and supplemented with 4.5 g L^−1^ Gelrite (Duchefa #G1101) or on half-strength MS medium (2.151 g L^−1^ Murashige and Skoog Basal Salt Mixture; Sigma-Aldrich #M5524), 2.56 mM MES hydrate, and 0.7% Phytoagar (Duchefa #P1003) in a 22°C day/18°C night cycle (8 h of light) at 120 µmol m^−2^ s^−1^. For experiments with *Serendipita indica*, roots of 9- to 10-d-old Arabidopsis plants were inoculated with 1 mL of a 500,000 chlamydospores mL^−1^ spore suspension per Petri dish. Control plants were treated with water containing 0.02% Tween 20 (mock). If not stated otherwise, plants were treated on plates with 1 mL per plate with a 1 µM solution of flg22 or Pep1 or with water as a control. For all experiments, flg22 and Pep1 peptides were used as described (Gómez-Gómez et al., 1999; Krol et al., 2010). All data are based on at least three independent biological experiments (if not specified otherwise).

### Measurement of Plant Growth Inhibition

For seedling growth inhibition assays, plants were grown on square Petri dishes for 10 d before inoculation with *S. indica* or mock treatment. After 3 d, plants were supplemented with 1 µM flg22, 1 µM Pep1, or water (control). Eleven days later, plant fresh weights were determined. For root growth inhibition assays, *pMYB51*:*NLS-3xmVENUS* and *pPER5*:*NLS-3xmVENUS* lines were grown on square Petri dishes for 10 d and treated with 1 mL per plate of 1 µM flg22 or 1 µM Pep1 solution in water. Control plants were treated with water (mock). Four days later, the plates were photographed and root lengths were measured using ImageJ software (https://imagej.net). If not stated otherwise, all biological experiments were done in triplicate and at least 12 plants per line per treatment were evaluated.

### Measurement of ROS Burst

Roots of 2-week-old plants were grown on solid ATS medium and treated with 1 µM flg22, 1 µM Pep1, or mock at 3 d after inoculation with *S. indica* or mock treatment. For ROS burst quantification, roots were cut into 1-cm-long pieces (10 mg per assay) and transferred to a luminol-based assay as described (Gómez-Gómez et al., 1999). Data were analyzed by Student’s *t* test.

### MAPK Protein and Phosphorylation Assay

Roots of 21-d-old Arabidopsis seedlings were harvested into liquid nitrogen 10, 30, and 60 min after immune elicitor treatment. Total protein was extracted after grinding and homogenizing the material in protein extraction buffer containing 15 mM Tris-HCl (pH 7.8), 25 mM NaCl, 75 mM EGTA, 15 mM MgCl_2_, 10 mM Tween 20, 0.1% (v/v) PMSF, 0.5 mM leupeptin, 10 μg μL^−1^ aprotinin, 10 μg μL^−1^ glycerophosphate, 15 mM NaF, 1 mM Na_3_VO_4_, and 0.5 mM DTT. Thirty to 40 μg of total protein extract was subjected to SDS-PAGE. Following transfer to nitrocellulose membrane, the proteins were incubated with monoclonal mouse anti-phospho-p44/42 MAPK (Erk1/2, Thr202/Tyr204; 1:1,000 dilution) antibody (Cell Signaling Technology), anti-MPK6 (1:10,000), anti-MPK3 (1:5000), and anti-MPK4 (1:5000) antibodies (all Sigma-Aldrich). The antibodies were diluted in 5% BSA in Tris-buffered saline plus Tween 20. After replacing primary with secondary anti-rabbit IgG HRP-conjugated antibody (1:10,000; Sigma-Aldrich), the samples were incubated for 2 h at room temperature before signal detection using a Femto-ECL kit (Pierce) and Amersham Hyperfilm (GE Healthcare).

### Gene Expression Analysis Using qRT-PCR

For gene expression analyses of whole roots, root material was harvested 2 and 24 h after flg22, Pep1, or control treatment. TRIzol (Invitrogen) was used to extract total RNA. After DNase treatment, RNA was reverse transcribed into cDNA using a qScript cDNA synthesis kit (Quanta Biosciences), and 10 ng of cDNA was used as a template in qRT-PCR using SYBR Green JumpStart Taq ReadyMix (Sigma-Aldrich) and a Stratagene Mx3005P Real-time PCR Detection System (Agilent Technologies) following the manufacturer’s recommended protocol. The 2^−ΔCt^ method (Schmittgen and Livak, 2008) was used to determine the differential expression of marker genes for immunity activation, and the housekeeping genes *UBIQUITIN5* (*UBQ5*, AT3G62250) and *ELONGATION FACTOR1α* (*EF1α*, AT5G60390; for primer sequences, see Supplemental Table 5) were used for normalization. Data were analyzed using Student’s *t* test.

### Quantification of *S. indica* Colonization by qRT-PCR

Genomic DNA was isolated from roots using a Plant DNeasy Kit (Qiagen). A total of 40 ng of genomic DNA served as the template in qRT-PCR using SYBR Green JumpStart Taq ReadyMix (Sigma-Aldrich) in a Stratagene Mx3005P Real-time PCR Detection System (Agilent Technologies) following the manufacturer’s recommended protocol. The 2^−ΔCt^ method (Schmittgen and Livak, 2008) was used to determine the extent of fungal colonization by subtracting the raw cycle threshold values of *S. indica* internal transcribed spacer from those of Arabidopsis *UBQ5* (for primer sequences, see Supplemental Table 5). Differences in *S. indica* colonization were determined by Student’s *t* test.

### FACS

For FACS experiments, plants were grown for 12 d on square ATS plates and treated with 1 mL per plate of 1 µM solutions of flg22 or Pep1 peptide or water as a control for 1 h. Taking into account the time required for protoplast generation and cell sorting (together ∼1 h), the status of all sampled cells was 2 h after flg22 or Pep1 treatment. Briefly, whole roots were cut into pieces and incubated in protoplast solution (1.5% cellulase R10 [Duchefa Biochemie], 1.2% cellulase RS [Duchefa Biochemie], 0.2% macerozyme R10 [Duchefa Biochemie], and 0.12% pectinase [Sigma-Aldrich] in 600 mM mannitol, 2 mM MES hydrate, 10 mM KCl, 2 mM CaCl_2_, 2 mM MgCl_2_, and 0.1% BSA, pH 5.7; Walker et al., 2017) for 45 min. Protoplasts were filtered through 70-µm followed by 40-µm cell strainers, centrifuged at 300*g* for 3 min, resuspended in protoplast solution lacking cell wall-degrading enzymes, and subjected to FACS. Three independent biological experiments were performed for each marker line. GFP-expressing protoplasts were collected using a BD Influx cell sorter (BD Biosciences) following previously published protocols (Birnbaum et al., 2003; Gifford et al., 2008; Grønlund et al., 2012). The cell sorter was equipped with a 100-µm nozzle, and BD FACSFlow (BD Biosciences) was used as the sheath fluid. BD Accudrop Fluorescent Beads (BD Biosciences) were used prior to each experiment to optimize sorting settings. A pressure of 20 p.s.i. (sheath) and 21 to 21.5 p.s.i. (sample) was applied during the experiments. Drop frequency was set to 39.2 kHz, and event rate was generally kept at <4000 events s^−1^. GFP-expressing protoplasts were identified using a 488-nm argon laser, plotting the outcome of a 580/30 bandpass filter versus a 530/40 bandpass filter, to differentiate between green fluorescence and autofluorescence. Different cell populations were collected for microscopy in preexperiments to determine the presence of GFP-expressing protoplasts. As previously reported (Grønlund et al., 2012), these protoplasts were present in the high 530-nm/low 580-nm population. Sorting gates were set conservatively in subsequent experiments based on these observations (Supplemental Figure 10). For RNA extraction, GFP-expressing protoplasts were sorted into Qiagen RLT lysis buffer containing 1% (v/v) β-mercaptoethanol, mixed, and immediately frozen at −80°C. At least 10,000 GFP-expressing protoplasts were sorted per experiment and treatment condition. Sorting times were kept below 20 min.

### RNA Isolation, RNA-Seq Library Construction, and Sequencing

Total RNA was extracted from the samples using a Qiagen RNeasy Plant Mini Kit including on-column DNase treatment with a Qiagen DNase kit. A 6000 Pico Kit (Agilent Technologies) was used to check quantity and quality of the RNA on a Bioanalyzer 2100 (Agilent Technologies). Preparation of amplified cDNA from total RNA and RNA-seq library construction were performed using the Ovation RNA-seq System V2 and Ovation Ultralow Library Systems Kits (NuGEN Technologies), respectively, following standard protocols. Sequencing was performed by the High-Throughput Genomics Group at the Wellcome Trust Centre for Human Genetics on an Illumina HiSeq2500 System.

### RNA-Seq Quality Control and Read Mapping

For each sample, read quality was evaluated using FastQC software (Andrews, 2010). The paired-end libraries (2 × 100-bp reads) were mapped to the Arabidopsis TAIR10 genome using STAR (default parameters; Dobin et al., 2013). The reads mapping to exons were counted using LiBiNorm (Dyer et al., 2019; settings: -f bam -s no -i Parent -t mRNA) using an Araport 11 annotation GTF file (Ensembl release 39). On average, 43% of reads uniquely mapped to exons (full details are given in Supplemental Table 1). The quality of read mapping was assessed using the Integrative Genomics Viewer (Robinson et al., 2011). The quality of replicates was assessed by plotting read counts of samples against one another and assessing the dispersion and presence of any artifacts between samples. Due to preferential amplification in some samples, reads corresponding to rRNA and ribosomal proteins had to be removed for subsequent analyses (Supplemental Data Set 11). The mitochondrial and plastid chromosomes were also removed, as this work focused on nucleus-encoded genes. Principal component analysis was calculated using the R function prcomp and visualized using ggplot2 implemented in R. Fragments per kilobase per million values were calculated for genes remaining after filtering using exonic gene lengths from the Araport 11 annotation GTF file (the same GTF file used for HTSeq-count; Supplemental Data Set 12).

### Differential Gene Expression and Functional Analysis

Within each cell type, genes that were differentially expressed in response to each treatment (compared with mock) were identified using DESeq2 (Love et al., 2014). All counts (minus filtered genes described above) were normalized using DESeq2 (default parameters). The model for differential expression included the replicate information in the model matrix (∼batch + condition) in order to account for batch effects. Genes were defined as differentially expressed if the adjusted P value was less than 0.05. We included all statistically significant genes and did not use a threshold on the fold change to (1) avoid the use of an arbitrary threshold, especially as thresholds do not necessarily correlate with translation and/or biological function, and (2) maximize statistical power for downstream analyses such as GO term or motif enrichment analyses. In order to define cell identity genes, first we identified genes that were differentially expressed between all possible pairs of cell type mock-treated samples using DESeq2 (again accounting for batches in the model matrix; P < 0.05). We then defined cell identity genes as those that were significantly upregulated in one cell type compared with both other cell types. For example, to define epidermis identity genes, we first identified genes differentially upregulated compared with the pericycle and cortex separately, then we took the intersection of these two lists of genes to be the epidermis identity genes. The significance of the overlap between cell identity genes and published cell-specific gene sets (Bargmann et al., 2013; Supplemental Data Set 13) was tested using Fisher’s exact test. From the published data set, epidermis-specific genes were taken to be the union of those labeled “trichoblast” and “atrichoblast,” cortex genes were those labeled as “cortex,” and pericycle genes were labeled as the union of “phloem-pole pericycle” and “xylem-pole pericycle” genes (Supplemental Data Set 14). The fit of the DESeq2 model to our data was tested by plotting replicates against one another and overlaying the read counts of DEGs (Supplemental Figure 4). Subset analysis to determine cell type- and treatment-exclusive genes was performed in R using built-in set functions and the VennDiagram (Chen and Boutros, 2011) package. Proportional visualizations of three-set Venn diagrams were created using eulerAPE (Micallef and Rodgers, 2014), nonproportional Venn diagrams were created using the Venn function from the R packaged gplots, and the six-set Venn diagram was created using the interactive Venn tool by Bardou et al. (2014).

GO enrichment analysis was performed using the R package GOStats (Falcon and Gentleman, 2007) with an additional Benjamini-Hochberg multiple testing correction applied. To make the P values across cell types directly comparable, we equalized gene set sizes by taking the top *K* genes from the larger data set where *K* is the size of the smaller data set for flg22 upregulated genes (cortex set: 128 genes; Figure 2A), Pep1 upregulated genes (epidermis set: 365 genes; Figure 2B), and Pep1 downregulated genes (epidermis set: 337 genes; Figure 2B). For the cell identity genes, we took the top 512 genes (Figure 3E). For the figures, the top 5 nonredundant terms were shown, where redundant terms were defined as terms that corresponded to exactly the same sets of genes. These were not shown in the plots in order to maximize the scope of the top GO terms.

### PMET

A total of 113 motifs (in the form of letter-probability matrices) were obtained from microarray studies performed by Franco-Zorrilla et al. (2014). These motifs characterize the target sequence specificity of 63 plant TFs representing 25 families. To conduct accurate comparisons, we equalized gene set sizes by taking all of the cortex-specific flg22 upregulated genes (128 genes; from Figure 2A) and the top 120 most significantly upregulated epidermis-specific genes. For Pep1, we included all Pep1-induced/suppressed epidermis genes (365 and 337 genes, respectively; Figure 2B) and the equivalent top Pep1-induced/suppressed genes in the cortex. Promoter regions corresponding to 1000 bp upstream from the transcription start site were collected from the TAIR database (Lamesch et al., 2012; www.arabidopsis.org) for all nuclear genes (including transposable element genes and excluding genes on the mitochondrial or plastid chromosomes) in the Arabidopsis genome. For each motif and each promoter, the sequence was scanned for occurrences of the motif using FIMO (Grant et al., 2011), which assigns a probability score to each potential hit. In order to determine the number of hits to consider and to compute an overall score for motif presence in a promoter, we computed the geometric mean *p* of the top *k* FIMO probability scores for nonoverlapping hits and computed the binomial probability of observing at least *k* hits of probability *p* in a 1-kb promoter. The value of *k* minimizing the binomial probability was taken to indicate the most likely number of binding sites, and the *k* hits were recorded for subsequent analysis (1 ≤ *k* ≤ 5). For each motif, the promoters were ordered by increasing binomial probability, and the top *n* = 5000 promoters were considered as containing the motif. Homogeneously setting *n* to the same value for each motif lays the foundation for computing P values for motif pairs that are comparable (see below) and avoids biases brought about by motifs of varying specificity. The parameter *n* was set to the high value of 5000 for high sensitivity (rather than specificity), as stringency is introduced when the pairing of motifs is considered. The binomial probability of the *n*-th promoter was recorded for each motif as a threshold. For each pair of motifs and for each promoter containing both motifs, overlaps of recorded motif hits were identified and the total information content (IC) of the overlap (based on motifs) was calculated. Total IC across the overlapping positions (*i*) of one motif was calculated as

$$IC=\;\underset{i\in I}{\sum }\left(2+\;\underset{b\in B}{\sum }f_{b,i}\log_{2}f_{b,i}\;\right)$$

where $f_{b,i}$ corresponds to the probability of observing base *b* at position *i*, according to previous work by Franco-Zorrilla et. al. (2014), and where B={A,C,G,T} and *I* is the set of positions in the overlap. If the IC of the overlap for either motif exceeded 4 (indicating that highly conserved bases are part of the overlap), these hits were removed and the binomial probability was recalculated for the remaining hits. The process of overlap removal is, therefore, only dependent on the IC and not directly dependent on the length of the overlap.

If the recalculated scores were still below the recorded motif-specific threshold, the two motifs were considered to be colocalized in the promoter. Finally, gene sets of interest were tested for enrichment of paired motifs using a pairwise hypergeometric test based on the MATLAB function proposed by Meng et al. (2009). Hypergeometric P values were corrected for the number of motif pairs using a stringent Bonferroni correction (calculating the correction for each gene set separately). As a negative control, random gene sets were tested against the same statistical method, resulting in no significant P values. Corrected P < 0.05 values are considered significant. For each comparison of results made between conditions, the gene sets tested were of equal size to make P values comparable. To this end, gene set sizes were equalized by taking the top *K* genes from the larger gene set, where *K* is the size of the smaller gene set.

### Generation of LhG4/pOp6 Transactivation Lines to Study Promoter Activities

In order to assess the PMET results, we decided to validate two pairs of motifs: WRKY-KAN enrichment in epidermis-specific flg22-responsive genes and MYC-WRKY enrichment in cortex-specific Pep1-responsive genes. We used short native promoters of three candidates, all of which were expressed strongly in a cell type-specific manner. *WRKY45* and *PIRL2* were chosen as genes to validate WRKY-KAN enrichment and *AtM10* was chosen to validate MYC-WRKY enrichment. Native promoter fragments (300 to 550 bp) were chosen such that they contained at least three binding sites for each TF type (WRKY, KAN, or MYC) but were short enough to reduce complexity arising from the presence of other TF binding motifs known to be present based on prior motif results. In the mutant versions of the promoters, WRKY, KAN, or MYC TF binding sites were replaced by the random sequence GAACTT. For synthetic promoters, we stacked TF binding sites in quadruplicate, each separated by 6 bp of a random spacer sequence (GAACTT), in front of the *CaMV35S* minimal promoter (nucleotides −46 to +1; Bhullar et al., 2003). In all cases, the presence or absence of TF binding sites was confirmed using FIMO, and we made sure that the insertion of random sequences did not result in the prediction of new TF binding sites. Native promoters or their mutated versions as well as synthetic promoter fragments were synthesized (Integrated DNA Technologies) flanked by attb sites for recombination into the Gateway-compatible pBIN+-GW-LhG4 vector (Costa et al., 2014). The resulting pBIN+Promoter-LhG4 “effector” plasmids were transformed into a stable *pOp6*:*YFP* “activator” line (Costa et al., 2014) via *Agrobacterium tumefaciens*-mediated transformation (Chang et al., 1994). Transformants were selected on hygromycin-containing medium, and the presence of the effector constructs was confirmed by PCR. For all assays, plants were grown in vertical square Petri dishes on ATS medium for 7 d and treated with 1 mL per plate of 1 μM flg22 or Pep1 or water (mock). Two hours later, whole roots of ∼30 plants per line and treatment were harvested for expression analysis by qRT-PCR (with *UBQ5* and *EF1α* for normalization as described above) or whole seedlings were harvested for confocal laser-scanning microscopy. The 2^−ΔΔCt^ method was used to determine *YFP* expression in transactivation plants relative to Col-0. To quantify YFP fluorescence in the synthetic promoter lines, maximum projections of the YFP channel from Z-stacks taken from root segments were analyzed using the ImageJ software package (https://imagej.net), and mean gray values for each image were determined. One-way ANOVA with Bonferroni posthoc test was applied to determine significance levels between native and synthetic promoter lines (see the Supplemental File for ANOVA tables).

### Confocal Laser-Scanning Microscopy

All images were taken with a confocal laser-scanning microscope (Zeiss LSM 880 or Leica SP5). For all experiments, GFP was excited with a 488-nm laser line and detected between 500 and 545 nm, and YFP and VENUS were excited at 514 nm and detected between 520 and 560 nm. *pMYB51*:*NLS-3xmVENUS*, *pPER5*:*NLS-3xmVENUS*, *pGL2*:*GFP*, *pCORTEX*:*GFP*, and *E3754* lines were imaged 1 h after flg22, Pep1, or mock treatment. For immunity and cell type-specific marker lines, viable roots were stained in a 10 μg mL^−1^ propidium iodide solution. Propidium iodide was excited at 561 nm, and fluorescence was detected between 570 and 720 nm. Due to the short time frame (2 to 3 h after treatment) for imaging of LhG4/pOp6 transactivation lines, we used a SCRI Renaissance 2200 (SR2200)/ClearSee staining method (Kurihara et al., 2015; Musielak et al., 2015), which involves fixation in paraformaldehyde. For this, seedlings were incubated in SR2200 staining solution (Musielak et al., 2015) for 30 min, followed by a washing step with PBS and clearing for at least 2 d in ClearSee solution (Kurihara et al., 2015). SR2200-stained cell walls were imaged using a 405-nm laser line for excitation and a bandwidth between 410 and 480 nm for detection.

### Uploading to ePlant

A scalable vector graphics (SVG) image representing our experimental setup was generated using the open-source SVG editing program Inkscape (https://inkscape.org). Expression data summarized as fragments per kilobase per million reads mapped were data based on the Bio-Analytic Resource for Plant Biology, and a web service was created to return expression values for a given gene across all samples in JSON (Javascript object notation) format. An XML file for a new Root Immunity Elicitation ePlant view was created to permit mapping of sample names to specific SVG parts and incorporated into ePlant (Waese et al., 2017). The appropriate group tags in the SVG file were edited to correspond to those in the XML file. The view may be freely accessed with ePlant at http://bar.utoronto.ca/eplant/?ActiveSpecies=Arabidopsis%20thalianaandGenes=AT2G35980andActiveGene=AT2G35980andActiveView=RootImmunityElicitationView (view for NHL10).

### Accession Numbers

The following supplemental data sets have been submitted to the Gene Expression Omnibus and are available at https://www.ncbi.nlm.nih.gov/geo/query/acc.cgi?acc=GSE112960: paired-end raw .fastq files and raw counts produced using LiBiNorm (Dyer et al., 2019) as .txt files. The data generated and analyzed during this study are available from the corresponding author on reasonable request. PMET has been made available as a web-based tool at http://bar.utoronto.ca/index.html (linked to http://nero.wsbc.warwick.ac.uk/tools), and the PMET source code is available on GitHub at https://github.com/kate-wa/PMET-software.

### Supplemental Data

**Supplemental Figure 1.** The ability of *S. indica* to suppress flg22 but not Pep1-triggered immune responses in roots.
**Supplemental Figure 2.** flg22 and Pep1 activate immunity in different root zones.
**Supplemental Figure 3.** Workflow for FACS analyses and RNA-seq.
**Supplemental Figure 4.** Visual inspection of differential gene expression.
**Supplemental Figure 5.** Assessment of RNA-seq data quality.
**Supplemental Figure 6.** Number of genes expressed per treatment and cell type.
**Supplemental Figure 7.** Promoter activities of selected candidate genes confirm cell type-specific expression in planta.
**Supplemental Figure 8.** Deletions in predicted promoter motif pairs in native promoters abolish cell type-specific gene regulation in planta.
**Supplemental Figure 9.** Quantification of YFP signals in the synthetic promoter lines shown in Figure 7.
**Supplemental Figure 10.** Representative FACS dot plots of the output from the 580/30 nm vs. the 530/40 nm bandpass filters.
**Supplemental Table 1.** Alignment statistics table based on output from htseq-count.
**Supplemental Table 2.** Number of DEGs responding to flg22 and Pep1 in three root cell types.
**Supplemental Table 3.** Genes differentially expressed in all three cell types in response to both flg22 and Pep1.
**Supplemental Table 4.** Number of DEGs responding specifically to either flg22 or Pep1 in one or more cell types.
**Supplemental Table 5.** Primer sequences.
**Supplemental Data Set 1.** Lists of all differentially expressed genes in response to flg22 and Pep1 from DESeq2 output.
**Supplemental Data Set 2.** Lists of cell type-specific DEGs following flg22 or Pep1 treatment.
**Supplemental Data Set 3.** Lists of cell type-specific GO terms after flg22 and Pep1 treatment.
**Supplemental Data Set 4.** Lists of flg22- and Pep1-specific DEGs per cell type.
**Supplemental Data Set 5.** Lists of flg22- and Pep1-specific GO terms per cell type.
**Supplemental Data Set 6.** Lists of cell identity genes and enriched GO terms.
**Supplemental Data Set 7.** Lists of PTI genes within cell type-specific cell identity genes.
**Supplemental Data Set 8.** Paired-motif analysis results for cell identity genes.
**Supplemental Data Set 9.** Paired-motif analysis results for flg22 up-regulated genes.
**Supplemental Data Set 10.** Paired-motif analysis results for Pep1 up- and down-regulated genes.
**Supplemental Data Set 11.** Genes omitted from the analysis.
**Supplemental Data Set 12.** Fragments per kilobase per million (FPKM) matrix of filtered genes.
**Supplemental Data Set 13.** Lists of differentially expressed genes between mock-treated cell types.
**Supplemental Data Set 14.** List of cell-specific genes as published (Bargmann et al., 2013).
**Supplemental File.** ANOVA tables.

## DIVE Curated Terms

The following phenotypic, genotypic, and functional terms are of significance to the work described in this paper:

MYC2 Gramene: AT1G32640
MYC2 Araport: AT1G32640

WRKY18 Gramene: AT4G31800
WRKY18 Araport: AT4G31800

YAB5 Gramene: AT2G26580
YAB5 Araport: AT2G26580

UBQ5 Gramene: AT3G62250
UBQ5 Araport: AT3G62250

WRKY45 Gramene: AT3G01970
WRKY45 Araport: AT3G01970

PROPEP1 Gramene: AT5G64900
PROPEP1 Araport: AT5G64900

WRKY22 Gramene: AT4G01250
WRKY22 Araport: AT4G01250

WRKY33 Gramene: AT2G38470
WRKY33 Araport: AT2G38470

FLS2 Gramene: Solyc02g070890
FLS2 Araport: Solyc02g070890

PTI Gramene: Pattern triggered immunity
PTI Araport: Pattern triggered immunity

MAMP Gramene: Microbe-associated molecular pattern
MAMP Araport: Microbe-associated molecular pattern

KAN1 Gramene: AT5G16560
KAN1 Araport: AT5G16560

ATS, KAN4 Gramene: AT5G42630
ATS, KAN4 Araport: AT5G42630

## References

[bib1] Achard, P., Gusti, A., Cheminant, S., Alioua, M., Dhondt, S., Coppens, F., Beemster, G.T.S., Genschik, P. (2009). Gibberellin signaling controls cell proliferation rate in Arabidopsis. Curr. Biol. 19: 1188–1193. 19576768 10.1016/j.cub.2009.05.059

[bib2] Alexandratos, N., Bruinsma, J. (2012). World Agriculture Towards 2030/2050: The 2012 Revision.. (Rome: Food and Agriculture Organization of the United Nations).

[bib3] Andrews, S. (2010). FastQC: A quality control tool for high throughput sequence data. http://www.bioinformatics.babraham.ac.uk/projects/fastqc/: http://www.bioinformatics.babraham.ac.uk/projects/.

[bib4] Asai, T., Tena, G., Plotnikova, J., Willmann, M.R., Chiu, W.-L., Gomez-Gomez, L., Boller, T., Ausubel, F.M., Sheen, J. (2002). MAP kinase signalling cascade in Arabidopsis innate immunity. Nature 415: 977–983. 11875555 10.1038/415977a

[bib5] Bardou, P., Mariette, J., Escudié, F., Djemiel, C., Klopp, C. (2014). jvenn: An interactive Venn diagram viewer. BMC Bioinformatics 15: 293. 25176396 10.1186/1471-2105-15-293 PMC4261873

[bib6] Bargmann, B.O.R., Vanneste, S., Krouk, G., Nawy, T., Efroni, I., Shani, E., Choe, G., Friml, J., Bergmann, D.C., Estelle, M., Birnbaum, K.D. (2013). A map of cell type-specific auxin responses. Mol. Syst. Biol. 9: 688. 24022006 10.1038/msb.2013.40 PMC3792342

[bib7] Bartels, S., Boller, T. (2015). Quo vadis, Pep? Plant elicitor peptides at the crossroads of immunity, stress, and development. J. Exp. Bot. 66: 5183–5193. 25911744 10.1093/jxb/erv180

[bib8] Beck, M., Wyrsch, I., Strutt, J., Wimalasekera, R., Webb, A., Boller, T., Robatzek, S. (2014). Expression patterns of flagellin sensing 2 map to bacterial entry sites in plant shoots and roots. J. Exp. Bot. 65: 6487–6498. 25205577 10.1093/jxb/eru366 PMC4246182

[bib9] Berendsen, R.L., Pieterse, C.M.J., Bakker, P.A.H.M. (2012). The rhizosphere microbiome and plant health. Trends Plant Sci. 17: 478–486. 22564542 10.1016/j.tplants.2012.04.001

[bib10] Bhullar, S., Chakravarthy, S., Advani, S., Datta, S., Pental, D., Burma, P.K. (2003). Strategies for development of functionally equivalent promoters with minimum sequence homology for transgene expression in plants: Cis-elements in a novel DNA context versus domain swapping. Plant Physiol. 132: 988–998. 12805627 10.1104/pp.103.020602 PMC167037

[bib11] Birnbaum, K., Jung, J.W., Wang, J.Y., Lambert, G.M., Hirst, J.A., Galbraith, D.W., Benfey, P.N. (2005). Cell type-specific expression profiling in plants via cell sorting of protoplasts from fluorescent reporter lines. Nat. Methods 2: 615–619. 16170893 10.1038/nmeth0805-615

[bib12] Birnbaum, K., Shasha, D.E., Wang, J.Y., Jung, J.W., Lambert, G.M., Galbraith, D.W., Benfey, P.N. (2003). A gene expression map of the Arabidopsis root. Science 302: 1956–1960. 14671301 10.1126/science.1090022

[bib13] Birnbaum, K.D. (2018). Power in numbers: Single-cell RNA-seq strategies to dissect complex tissues. Annu. Rev. Genet. 52: 203–221. 30192636 10.1146/annurev-genet-120417-031247 PMC6314027

[bib14] Boller, T., Felix, G. (2009). A renaissance of elicitors: Perception of microbe-associated molecular patterns and danger signals by pattern-recognition receptors. Annu. Rev. Plant Biol. 60: 379–406. 19400727 10.1146/annurev.arplant.57.032905.105346

[bib15] Brady, S.M., Orlando, D.A., Lee, J.Y., Wang, J.Y., Koch, J., Dinneny, J.R., Mace, D., Ohler, U., Benfey, P.N. (2007). A high-resolution root spatiotemporal map reveals dominant expression patterns. Science 318: 801–806. 17975066 10.1126/science.1146265

[bib16] Bulgarelli, D., Schlaeppi, K., Spaepen, S., Ver Loren van Themaat, E., Schulze-Lefert, P. (2013). Structure and functions of the bacterial microbiota of plants. Annu. Rev. Plant Biol. 64: 807–838. 23373698 10.1146/annurev-arplant-050312-120106

[bib17] Chang, S.S., Park, S.K., Kim, B.C., Kang, B.J., Kim, D.U., Nam, H.G. (1994). Stable genetic transformation of *Arabidopsis thaliana* by Agrobacterium inoculation in planta. Plant J. 5: 551–558.

[bib18] Chen, H., Boutros, P.C. (2011). VennDiagram: A package for the generation of highly-customizable Venn and Euler diagrams in R. BMC Bioinformatics 12: 35. 21269502 10.1186/1471-2105-12-35 PMC3041657

[bib19] Clark, N.M., Hinde, E., Winter, C.M., Fisher, A.P., Crosti, G., Blilou, I., Gratton, E., Benfey, P.N., Sozzani, R. (2016). Tracking transcription factor mobility and interaction in Arabidopsis roots with fluorescence correlation spectroscopy. eLife 5: e14770. 27288545 10.7554/eLife.14770 PMC4946880

[bib20] Cook, D.E., Mesarich, C.H., Thomma, B.P.H.J. (2015). Understanding plant immunity as a surveillance system to detect invasion. Annu. Rev. Phytopathol. 53: 541–563. 26047564 10.1146/annurev-phyto-080614-120114

[bib21] Costa, L.M., (2014). Central cell-derived peptides regulate early embryo patterning in flowering plants. Science 344: 168–172. 24723605 10.1126/science.1243005

[bib22] Craft, J., Samalova, M., Baroux, C., Townley, H., Martinez, A., Jepson, I., Tsiantis, M., Moore, I. (2005). New pOp/LhG4 vectors for stringent glucocorticoid-dependent transgene expression in Arabidopsis. Plant J. 41: 899–918. 15743453 10.1111/j.1365-313X.2005.02342.x

[bib23] Delgado-Baquerizo, M., Guerra, C.A., Cano-Díaz, C., Egidi, E., Wang, J.T., Eisenhauer, N., Singh, B.K., Maestre, F.T. (2020). The proportion of soil-borne pathogens increases with warming at the global scale. Nat. Clim. Chang. 10: 550–554.

[bib24] Denyer, T., Ma, X., Klesen, S., Scacchi, E., Nieselt, K., Timmermans, M.C.P. (2019). Spatiotemporal developmental trajectories in the Arabidopsis root revealed using high-throughput single-cell RNA sequencing. Dev. Cell 48: 840–852.e5. 30913408 10.1016/j.devcel.2019.02.022

[bib25] De Smet, I. (2012). Lateral root initiation: One step at a time. New Phytol. 193: 867–873. 22403823 10.1111/j.1469-8137.2011.03996.x

[bib26] Dinneny, J.R., Long, T.A., Wang, J.Y., Jung, J.W., Mace, D., Pointer, S., Barron, C., Brady, S.M., Schiefelbein, J., Benfey, P.N. (2008). Cell identity mediates the response of Arabidopsis roots to abiotic stress. Science 320: 942–945. 18436742 10.1126/science.1153795

[bib27] Dobin, A., Davis, C.A., Schlesinger, F., Drenkow, J., Zaleski, C., Jha, S., Batut, P., Chaisson, M., Gingeras, T.R. (2013). STAR: Ultrafast universal RNA-seq aligner. Bioinformatics 29: 15–21. 23104886 10.1093/bioinformatics/bts635 PMC3530905

[bib28] Dolan, L., Janmaat, K., Willemsen, V., Linstead, P., Poethig, S., Roberts, K., Scheres, B. (1993). Cellular organisation of the *Arabidopsis thaliana* root. Development 119: 71–84. 8275865 10.1242/dev.119.1.71

[bib29] Dombrecht, B., Xue, G.P., Sprague, S.J., Kirkegaard, J.A., Ross, J.J., Reid, J.B., Fitt, G.P., Sewelam, N., Schenk, P.M., Manners, J.M., Kazan, K. (2007). MYC2 differentially modulates diverse jasmonate-dependent functions in Arabidopsis. Plant Cell 19: 2225–2245. 17616737 10.1105/tpc.106.048017 PMC1955694

[bib30] Du, Y., Scheres, B. (2018). Lateral root formation and the multiple roles of auxin. J. Exp. Bot. 69: 155–167. 28992266 10.1093/jxb/erx223

[bib31] Dyer, N.P., Shahrezaei, V., Hebenstreit, D. (2019). LiBiNorm: An htseq-count analogue with improved normalisation of Smart-seq2 data and library preparation diagnostics. PeerJ 7: e6222. 30740268 10.7717/peerj.6222 PMC6366399

[bib32] Ezer, D., Zabet, N.R., Adryan, B. (2014). Homotypic clusters of transcription factor binding sites: A model system for understanding the physical mechanics of gene expression. Comput. Struct. Biotechnol. J. 10: 63–69. 25349675 10.1016/j.csbj.2014.07.005 PMC4204428

[bib33] Falcon, S., Gentleman, R. (2007). Using GOstats to test gene lists for GO term association. Bioinformatics 23: 257–258. 17098774 10.1093/bioinformatics/btl567

[bib34] Felix, G., Duran, J.D., Volko, S., Boller, T. (1999). Plants have a sensitive perception system for the most conserved domain of bacterial flagellin. Plant J. 18: 265–276. 10377992 10.1046/j.1365-313x.1999.00265.x

[bib35] Fernández-Calvo, P., (2011). The Arabidopsis bHLH transcription factors MYC3 and MYC4 are targets of JAZ repressors and act additively with MYC2 in the activation of jasmonate responses. Plant Cell 23: 701–715. 21335373 10.1105/tpc.110.080788 PMC3077776

[bib36] Flury, P., Klauser, D., Schulze, B., Boller, T., Bartels, S. (2013). The anticipation of danger: Microbe-associated molecular pattern perception enhances AtPep-triggered oxidative burst. Plant Physiol. 161: 2023–2035. 23400703 10.1104/pp.113.216077 PMC3613473

[bib37] Franco-Zorrilla, J.M., López-Vidriero, I., Carrasco, J.L., Godoy, M., Vera, P., Solano, R. (2014). DNA-binding specificities of plant transcription factors and their potential to define target genes. Proc. Natl. Acad. Sci. USA 111: 2367–2372. 24477691 10.1073/pnas.1316278111 PMC3926073

[bib38] Geng, Y., Wu, R., Wee, C.W., Xie, F., Wei, X., Chan, P.M.Y., Tham, C., Duan, L., Dinneny, J.R. (2013). A spatio-temporal understanding of growth regulation during the salt stress response in Arabidopsis. Plant Cell 25: 2132–2154. 23898029 10.1105/tpc.113.112896 PMC3723617

[bib39] Gewin, V. (2010). Food: An underground revolution. Nature 466: 552–553. 20671689 10.1038/466552a

[bib40] Gifford, M.L., Banta, J.A., Katari, M.S., Hulsmans, J., Chen, L., Ristova, D., Tranchina, D., Purugganan, M.D., Coruzzi, G.M., Birnbaum, K.D. (2013). Plasticity regulators modulate specific root traits in discrete nitrogen environments. PLoS Genet. 9: e1003760. 24039603 10.1371/journal.pgen.1003760 PMC3764102

[bib41] Gifford, M.L., Dean, A., Gutierrez, R.A., Coruzzi, G.M., Birnbaum, K.D. (2008). Cell-specific nitrogen responses mediate developmental plasticity. Proc. Natl. Acad. Sci. USA 105: 803–808. 18180456 10.1073/pnas.0709559105 PMC2206617

[bib42] Gómez-Gómez, L., Felix, G., Boller, T. (1999). A single locus determines sensitivity to bacterial flagellin in *Arabidopsis thaliana*. Plant J. 18: 277–284. 10377993 10.1046/j.1365-313x.1999.00451.x

[bib43] Gonneau, M., (2018). Receptor kinase THESEUS1 is a rapid alkalinization factor 34 receptor in Arabidopsis. Curr. Biol. 28: 2452–2458.e4. 30057301 10.1016/j.cub.2018.05.075

[bib44] Grant, C.E., Bailey, T.L., Noble, W.S. (2011). FIMO: Scanning for occurrences of a given motif. Bioinformatics 27: 1017–1018. 21330290 10.1093/bioinformatics/btr064 PMC3065696

[bib45] Grønlund, J.T., Eyres, A., Kumar, S., Buchanan-Wollaston, V., Gifford, M.L. (2012). Cell specific analysis of Arabidopsis leaves using fluorescence activated cell sorting. J. Vis. Exp. 68: e4214. 10.3791/4214 PMC3490320 23070217

[bib46] Halfon, M.S., Carmena, A., Gisselbrecht, S., Sackerson, C.M., Jiménez, F., Baylies, M.K., Michelson, A.M. (2000). Ras pathway specificity is determined by the integration of multiple signal-activated and tissue-restricted transcription factors. Cell 103: 63–74. 11051548 10.1016/s0092-8674(00)00105-7

[bib47] Haruta, M., Sabat, G., Stecker, K., Minkoff, B.B., Sussman, M.R. (2014). A peptide hormone and its receptor protein kinase regulate plant cell expansion. Science 343: 408–411. 24458638 10.1126/science.1244454 PMC4672726

[bib48] Hawker, N.P.N., Bowman, J.L.J. (2004). Roles for Class III HD-Zip and KANADI genes in Arabidopsis root development. Plant Physiol. 135: 2261–2270. 15286295 10.1104/pp.104.040196 PMC520795

[bib49] Huffaker, A., Pearce, G., Ryan, C.A. (2006). An endogenous peptide signal in Arabidopsis activates components of the innate immune response. Proc. Natl. Acad. Sci. USA 103: 10098–10103. 16785434 10.1073/pnas.0603727103 PMC1502512

[bib50] Hur, Y.-S., (2015). Arabidopsis thaliana homeobox 12 (ATHB12), a homeodomain-leucine zipper protein, regulates leaf growth by promoting cell expansion and endoreduplication. New Phytol. 205: 316–328. 25187356 10.1111/nph.12998

[bib51] Ilegems, M., Douet, V., Meylan-Bettex, M., Uyttewaal, M., Brand, L., Bowman, J.L., Stieger, P.A. (2010). Interplay of auxin, KANADI and Class III HD-ZIP transcription factors in vascular tissue formation. Development 137: 975–984. 20179097 10.1242/dev.047662

[bib52] Iyer-Pascuzzi, A.S., Jackson, T., Cui, H., Petricka, J.J., Busch, W., Tsukagoshi, H., Benfey, P.N. (2011). Cell identity regulators link development and stress responses in the Arabidopsis root. Dev. Cell 21: 770–782. 22014526 10.1016/j.devcel.2011.09.009 PMC3204215

[bib53] Jacobs, S., Zechmann, B., Molitor, A., Trujillo, M., Petutschnig, E., Lipka, V., Kogel, K.-H., Schäfer, P. (2011). Broad-spectrum suppression of innate immunity is required for colonization of Arabidopsis roots by the fungus *Piriformospora indica*. Plant Physiol. 156: 726–740. 21474434 10.1104/pp.111.176446 PMC3177271

[bib54] Jean-Baptiste, K., McFaline-Figueroa, J.L., Alexandre, C.M., Dorrity, M.W., Saunders, L., Bubb, K.L., Trapnell, C., Fields, S., Queitsch, C., Cuperus, J.T. (2019). Dynamics of gene expression in single root cells of *Arabidopsis thaliana*. Plant Cell 31: 993–1011. 30923229 10.1105/tpc.18.00785 PMC8516002

[bib55] Jones, J.D.G., Dangl, J.L. (2006). The plant immune system. Nature 444: 323–329. 17108957 10.1038/nature05286

[bib56] Jourquin, J., Fukaki, H., Beeckman, T. (2020). Peptide-receptor signaling controls lateral root development. Plant Physiol. 182: 1645–1656. 31862841 10.1104/pp.19.01317 PMC7140930

[bib57] Junion, G., Spivakov, M., Girardot, C., Braun, M., Gustafson, E.H., Birney, E., Furlong, E.E.M. (2012). A transcription factor collective defines cardiac cell fate and reflects lineage history. Cell 148: 473–486. 22304916 10.1016/j.cell.2012.01.030

[bib58] Krol, E., Mentzel, T., Chinchilla, D., Boller, T., Felix, G., Kemmerling, B., Postel, S., Arents, M., Jeworutzki, E., Al-Rasheid, K.A.S., Becker, D., Hedrich, R. (2010). Perception of the Arabidopsis danger signal peptide 1 involves the pattern recognition receptor AtPEPR1 and its close homologue AtPEPR2. J. Biol. Chem. 285: 13471–13479. 20200150 10.1074/jbc.M109.097394 PMC2859507

[bib59] Kurihara, D., Mizuta, Y., Sato, Y., Higashiyama, T. (2015). ClearSee: A rapid optical clearing reagent for whole-plant fluorescence imaging. Development 142: 4168–4179. 26493404 10.1242/dev.127613 PMC4712841

[bib60] Lamesch, P., (2012). The Arabidopsis Information Resource (TAIR): Improved gene annotation and new tools. Nucleic Acids Res. 40: D1202–D1210. 22140109 10.1093/nar/gkr1090 PMC3245047

[bib61] Lareen, A., Burton, F., Schäfer, P. (2016). Plant root-microbe communication in shaping root microbiomes. Plant Mol. Biol. 90: 575–587. 26729479 10.1007/s11103-015-0417-8 PMC4819777

[bib62] Lewis, L.A., (2015). Transcriptional dynamics driving MAMP-triggered immunity and pathogen effector-mediated immunosuppression in Arabidopsis leaves following infection with *Pseudomonas syringae* pv *tomato* DC3000. Plant Cell 27: 3038–3064. 26566919 10.1105/tpc.15.00471 PMC4682296

[bib63] Lin, Q., Ohashi, Y., Kato, M., Tsuge, T., Gu, H., Qu, L.-J., Aoyama, T. (2015). GLABRA2 directly suppresses basic helix-loop-helix transcription factor genes with diverse functions in root hair development. Plant Cell 27: 2894–2906. 26486447 10.1105/tpc.15.00607 PMC4637992

[bib64] Lincoln, C., Britton, J.H., Estelle, M. (1990). Growth and development of the *axr1* mutants of Arabidopsis. Plant Cell 2: 1071–1080. 1983791 10.1105/tpc.2.11.1071 PMC159955

[bib65] Liu, Z., Wu, Y., Yang, F., Zhang, Y., Chen, S., Xie, Q., Tian, X., Zhou, J.-M. (2013). BIK1 interacts with PEPRs to mediate ethylene-induced immunity. Proc. Natl. Acad. Sci. USA 110: 6205–6210. 23431184 10.1073/pnas.1215543110 PMC3625333

[bib66] Lori, M., van Verk, M.C., Hander, T., Schatowitz, H., Klauser, D., Flury, P., Gehring, C.A., Boller, T., Bartels, S. (2015). Evolutionary divergence of the plant elicitor peptides (Peps) and their receptors: Interfamily incompatibility of perception but compatibility of downstream signalling. J. Exp. Bot. 66: 5315–5325. 26002971 10.1093/jxb/erv236 PMC4526913

[bib67] Love, M.I., Huber, W., Anders, S. (2014). Moderated estimation of fold change and dispersion for RNA-seq data with DESeq2. Genome Biol. 15: 550. 25516281 10.1186/s13059-014-0550-8 PMC4302049

[bib68] Masucci, J.D., Rerie, W.G., Foreman, D.R., Zhang, M., Galway, M.E., Marks, M.D., Schiefelbein, J.W. (1996). The homeobox gene GLABRA2 is required for position-dependent cell differentiation in the root epidermis of *Arabidopsis thaliana*. Development 122: 1253–1260. 8620852 10.1242/dev.122.4.1253

[bib69] Matsushita, A., Furumoto, T., Ishida, S., Takahashi, Y. (2007). AGF1, an AT-hook protein, is necessary for the negative feedback of AtGA3ox1 encoding GA 3-oxidase. Plant Physiol. 143: 1152–1162. 17277098 10.1104/pp.106.093542 PMC1820926

[bib70] McAbee, J.M., Hill, T.A., Skinner, D.J., Izhaki, A., Hauser, B.A., Meister, R.J., Venugopala Reddy, G., Meyerowitz, E.M., Bowman, J.L., Gasser, C.S. (2006). ABERRANT TESTA SHAPE encodes a KANADI family member, linking polarity determination to separation and growth of Arabidopsis ovule integuments. Plant J. 46: 522–531. 16623911 10.1111/j.1365-313X.2006.02717.x

[bib71] Meng, J., Gao, S.-J., Huang, Y. (2009). Enrichment constrained time-dependent clustering analysis for finding meaningful temporal transcription modules. Bioinformatics 25: 1521–1527. 19351618 10.1093/bioinformatics/btp235 PMC2687989

[bib72] Miao, Z.-Q., Zhao, P.-X., Mao, J., Yu, L., Yuan, Y., Tang, H., Liu, Z.-B., Xiang, C. (2018). HOMEOBOX PROTEIN52 mediates the crosstalk between ethylene and auxin signaling during primary root elongation by modulating auxin transport-related gene expression. Plant Cell 30: 2761–2778. 30333147 10.1105/tpc.18.00584 PMC6305987

[bib73] Micallef, L., Rodgers, P. (2014). eulerAPE: Drawing area-proportional 3-Venn diagrams using ellipses. PLoS One 9: e101717. 25032825 10.1371/journal.pone.0101717 PMC4102485

[bib74] Millet, Y.A., Danna, C.H., Clay, N.K., Songnuan, W., Simon, M.D., Werck-Reichhart, D., Ausubel, F.M. (2010). Innate immune responses activated in Arabidopsis roots by microbe-associated molecular patterns. Plant Cell 22: 973–990. 20348432 10.1105/tpc.109.069658 PMC2861455

[bib75] Miyashima, S., (2019). Mobile PEAR transcription factors integrate positional cues to prime cambial growth. Nature 565: 490–494. 30626969 10.1038/s41586-018-0839-y PMC7617008

[bib76] Moore, I., Gälweiler, L., Grosskopf, D., Schell, J., Palme, K. (1998). A transcription activation system for regulated gene expression in transgenic plants. Proc. Natl. Acad. Sci. USA 95: 376–381. 9419383 10.1073/pnas.95.1.376 PMC18229

[bib77] Murphy, E., (2016). RALFL34 regulates formative cell divisions in Arabidopsis pericycle during lateral root initiation. J. Exp. Bot. 67: 4863–4875. 27521602 10.1093/jxb/erw281 PMC4983113

[bib78] Musielak, T.J., Schenkel, L., Kolb, M., Henschen, A., Bayer, M. (2015). A simple and versatile cell wall staining protocol to study plant reproduction. Plant Reprod. 28: 161–169. 26454832 10.1007/s00497-015-0267-1 PMC4623088

[bib79] Nakajima, K., Sena, G., Nawy, T., Benfey, P.N. (2001). Intercellular movement of the putative transcription factor SHR in root patterning. Nature 413: 307–311. 11565032 10.1038/35095061

[bib80] Ortiz-Morea, F.A., (2016). Danger-associated peptide signaling in Arabidopsis requires clathrin. Proc. Natl. Acad. Sci. USA 113: 11028–11033. 27651494 10.1073/pnas.1605588113 PMC5047203

[bib81] Pandey, S.P., Somssich, I.E. (2009). The role of WRKY transcription factors in plant immunity. Plant Physiol. 150: 1648–1655. 19420325 10.1104/pp.109.138990 PMC2719123

[bib82] Panth, M., Hassler, S.C., Baysal-Gurel, F. (2020). Methods for management of soilborne diseases in crop production. Agriculture 10: 16.

[bib83] Parizot, B., Roberts, I., Raes, J., Beeckman, T., De Smet, I. (2012). In silico analyses of pericycle cell populations reinforce their relation with associated vasculature in Arabidopsis. Philos. Trans. R. Soc. Lond. B Biol. Sci. 367: 1479–1488. 22527390 10.1098/rstb.2011.0227 PMC3321678

[bib84] Poncini, L., Wyrsch, I., Dénervaud Tendon, V., Vorley, T., Boller, T., Geldner, N., Métraux, J.-P., Lehmann, S. (2017). In roots of *Arabidopsis thaliana*, the damage-associated molecular pattern AtPep1 is a stronger elicitor of immune signalling than flg22 or the chitin heptamer. PLoS One 12: e0185808. 28973025 10.1371/journal.pone.0185808 PMC5626561

[bib85] Qi, Z., Verma, R., Gehring, C., Yamaguchi, Y., Zhao, Y., Ryan, C.A., Berkowitz, G.A. (2010). Ca^2+^ signaling by plant *Arabidopsis thaliana* Pep peptides depends on AtPepR1, a receptor with guanylyl cyclase activity, and cGMP-activated Ca^2+^ channels. Proc. Natl. Acad. Sci. USA 107: 21193–21198. 21088220 10.1073/pnas.1000191107 PMC3000296

[bib86] Rich-Griffin, C., Stechemesser, A., Finch, J., Lucas, E., Ott, S., Schäfer, P. (2020). Single-cell transcriptomics: A high-resolution avenue for plant functional genomics. Trends Plant Sci. 25: 186–197. 31780334 10.1016/j.tplants.2019.10.008

[bib87] Robinson, J.T., Thorvaldsdóttir, H., Winckler, W., Guttman, M., Lander, E.S., Getz, G., Mesirov, J.P. (2011). Integrative genomics viewer. Nat. Biotechnol. 29: 24–26. 21221095 10.1038/nbt.1754 PMC3346182

[bib88] Rodríguez-Martínez, J.A., Reinke, A.W., Bhimsaria, D., Keating, A.E., Ansari, A.Z. (2017). Combinatorial bZIP dimers display complex DNA-binding specificity landscapes. eLife 6: e19272. 28186491 10.7554/eLife.19272 PMC5349851

[bib89] Ross-Elliott, T.J., (2017). Phloem unloading in Arabidopsis roots is convective and regulated by the phloem-pole pericycle. eLife 6: e24125. 28230527 10.7554/eLife.24125 PMC5365319

[bib90] Rushton, P.J., Somssich, I.E., Ringler, P., Shen, Q.J. (2010). WRKY transcription factors. Trends Plant Sci. 15: 247–258. 20304701 10.1016/j.tplants.2010.02.006

[bib91] Ryan, C.A., Huffaker, A., Yamaguchi, Y. (2007). New insights into innate immunity in Arabidopsis. Cell. Microbiol. 9: 1902–1908. 17593247 10.1111/j.1462-5822.2007.00991.x

[bib92] Ryu, K.H., Huang, L., Kang, H.M., Schiefelbein, J. (2019). Single-cell RNA sequencing resolves molecular relationships among individual plant cells. Plant Physiol. 179: 1444–1456. 30718350 10.1104/pp.18.01482 PMC6446759

[bib93] Sabatini, S., Heidstra, R., Wildwater, M., Scheres, B. (2003). SCARECROW is involved in positioning the stem cell niche in the Arabidopsis root meristem. Genes Dev. 17: 354–358. 12569126 10.1101/gad.252503 PMC195985

[bib94] Sarojam, R., Sappl, P.G., Goldshmidt, A., Efroni, I., Floyd, S.K., Eshed, Y., Bowman, J.L. (2010). Differentiating Arabidopsis shoots from leaves by combined YABBY activities. Plant Cell 22: 2113–2130. 20628155 10.1105/tpc.110.075853 PMC2929102

[bib95] Schang, A.L., (2018). Discordant perturbations of transcriptome and epigenome landscapes highlight dual roles of proinflammatory players in normal and IL1B-compromised OPC maturation trajectory in a prenatal model of diffuse white matter injury. bioRxiv 411702.

[bib96] Schmittgen, T.D., Livak, K.J. (2008). Analyzing real-time PCR data by the comparative C(T) method. Nat. Protoc. 3: 1101–1108. 18546601 10.1038/nprot.2008.73

[bib97] Schulze, B., Mentzel, T., Jehle, A.K., Mueller, K., Beeler, S., Boller, T., Felix, G., Chinchilla, D. (2010). Rapid heteromerization and phosphorylation of ligand-activated plant transmembrane receptors and their associated kinase BAK1. J. Biol. Chem. 285: 9444–9451. 20103591 10.1074/jbc.M109.096842 PMC2843194

[bib98] Schweizer, F., Fernández-Calvo, P., Zander, M., Diez-Diaz, M., Fonseca, S., Glauser, G., Lewsey, M.G., Ecker, J.R., Solano, R., Reymond, P. (2013). Arabidopsis basic helix-loop-helix transcription factors MYC2, MYC3, and MYC4 regulate glucosinolate biosynthesis, insect performance, and feeding behavior. Plant Cell 25: 3117–3132. 23943862 10.1105/tpc.113.115139 PMC3784603

[bib99] Stegmann, M., Monaghan, J., Smakowska-Luzan, E., Rovenich, H., Lehner, A., Holton, N., Belkhadir, Y., Zipfel, C. (2017). The receptor kinase FER is a RALF-regulated scaffold controlling plant immune signaling. Science 355: 287–289. 28104890 10.1126/science.aal2541

[bib100] Stringlis, I.A., Proietti, S., Hickman, R., Van Verk, M.C., Zamioudis, C., Pieterse, C.M.J. (2018). Root transcriptional dynamics induced by beneficial rhizobacteria and microbial immune elicitors reveal signatures of adaptation to mutualists. Plant J. 93: 166–180. 29024173 10.1111/tpj.13741 PMC5765484

[bib101] Takano, J., Noguchi, K., Yasumori, M., Kobayashi, M., Gajdos, Z., Miwa, K., Hayashi, H., Yoneyama, T., Fujiwara, T. (2002). Arabidopsis boron transporter for xylem loading. Nature 420: 337–340. 12447444 10.1038/nature01139

[bib102] Tintor, N., Ross, A., Kanehara, K., Yamada, K., Fan, L., Kemmerling, B., Nürnberger, T., Tsuda, K., Saijo, Y. (2013). Layered pattern receptor signaling via ethylene and endogenous elicitor peptides during Arabidopsis immunity to bacterial infection. Proc. Natl. Acad. Sci. USA 110: 6211–6216. 23431187 10.1073/pnas.1216780110 PMC3625345

[bib103] van den Berg, C., Willemsen, V., Hage, W., Weisbeek, P., Scheres, B. (1995). Cell fate in the Arabidopsis root meristem determined by directional signalling. Nature 378: 62–65. 7477287 10.1038/378062a0

[bib104] Vandepoele, K., Casneuf, T., Van de Peer, Y. (2006). Identification of novel regulatory modules in dicotyledonous plants using expression data and comparative genomics. Genome Biol. 7: R103. 17090307 10.1186/gb-2006-7-11-r103 PMC1794593

[bib105] Van de Velde, J., Heyndrickx, K.S., Vandepoele, K. (2014). Inference of transcriptional networks in Arabidopsis through conserved noncoding sequence analysis. Plant Cell 26: 2729–2745. 24989046 10.1105/tpc.114.127001 PMC4145110

[bib106] Waese, J., (2017). ePlant: Visualizing and exploring multiple levels of data for hypothesis generation in plant biology. Plant Cell 29: 1806–1821. 28808136 10.1105/tpc.17.00073 PMC5590499

[bib107] Walker, L., (2017). Changes in gene expression in space and time orchestrate environmentally mediated shaping of root architecture. Plant Cell 29: 2393–2412. 28893852 10.1105/tpc.16.00961 PMC5774560

[bib108] Wendrich, J.R., Möller, B.K., Li, S., Saiga, S., Sozzani, R., Benfey, P.N., De Rybel, B., Weijers, D. (2017). Framework for gradual progression of cell ontogeny in the *Arabidopsis* root meristem. Proc. Natl. Acad. Sci. USA 114: E8922–E8929. 28973915 10.1073/pnas.1707400114 PMC5651754

[bib109] Wyrsch, I., Domínguez-Ferreras, A., Geldner, N., Boller, T. (2015). Tissue-specific FLAGELLIN-SENSING 2 (FLS2) expression in roots restores immune responses in Arabidopsis fls2 mutants. New Phytol. 206: 774–784. 25627577 10.1111/nph.13280

[bib110] Xu, X.M., Wang, J., Xuan, Z., Goldshmidt, A., Borrill, P.G.M., Hariharan, N., Kim, J.Y., Jackson, D. (2011). Chaperonins facilitate KNOTTED1 cell-to-cell trafficking and stem cell function. Science 333: 1141–1144. 21868675 10.1126/science.1205727

[bib111] Yamada, K., Yamashita-Yamada, M., Hirase, T., Fujiwara, T., Tsuda, K., Hiruma, K., Saijo, Y. (2016). Danger peptide receptor signaling in plants ensures basal immunity upon pathogen-induced depletion of BAK1. EMBO J. 35: 46–61. 26574534 10.15252/embj.201591807 PMC4718002

[bib112] Yamaguchi, Y., Huffaker, A. (2011). Endogenous peptide elicitors in higher plants. Curr. Opin. Plant Biol. 14: 351–357. 21636314 10.1016/j.pbi.2011.05.001

[bib113] Yamaguchi, Y., Huffaker, A., Bryan, A.C., Tax, F.E., Ryan, C.A. (2010). PEPR2 is a second receptor for the Pep1 and Pep2 peptides and contributes to defense responses in Arabidopsis. Plant Cell 22: 508–522. 20179141 10.1105/tpc.109.068874 PMC2845411

[bib114] Zhang, T.Q., Xu, Z.G., Shang, G.D., Wang, J.W. (2019). A single-cell RNA sequencing profiles the developmental landscape of Arabidopsis root. Mol. Plant 12: 648–660. 31004836 10.1016/j.molp.2019.04.004

[bib115] Zhou, F., Emonet, A., Dénervaud Tendon, V., Marhavy, P., Wu, D., Lahaye, T., Geldner, N. (2020). Co-incidence of damage and microbial patterns controls localized immune responses in roots. Cell 180: 440–453.e18. 32032516 10.1016/j.cell.2020.01.013 PMC7042715

[bib116] Zipfel, C., Robatzek, S., Navarro, L., Oakeley, E.J., Jones, J.D.G., Felix, G., Boller, T. (2004). Bacterial disease resistance in Arabidopsis through flagellin perception. Nature 428: 764–767. 15085136 10.1038/nature02485

